# A computational study of CH_4_ storage in porous framework materials with metalated linkers: connecting the atomistic character of CH_4_ binding sites to usable capacity†

**DOI:** 10.1039/c6sc00529b

**Published:** 2016-03-29

**Authors:** Ehud Tsivion, Jarad A. Mason, Miguel. I. Gonzalez, Jeffrey R. Long, Martin Head-Gordon

**Affiliations:** a Materials Sciences Division, Lawrence Berkeley National Laboratory Berkeley California 94720 USA; b Chemical Sciences Division, Lawrence Berkeley National Laboratory Berkeley California 94720 USA; c Department of Chemistry, University of California Berkeley California 94720 USA mhg@cchem.berkeley.edu; d Department of Chemical and Biomolecular Engineering, University of California Berkeley California 94720 USA

## Abstract

To store natural gas (NG) inexpensively at adequate densities for use as a fuel in the transportation sector, new porous materials are being developed. This work uses computational methods to explore strategies for improving the usable methane storage capacity of adsorbents, including metal–organic frameworks (MOFs), that feature open-metal sites incorporated into their structure by postsynthetic modification. The adsorption of CH_4_ on several open-metal sites is studied by calculating geometries and adsorption energies and analyzing the relevant interaction factors. Approximate site-specific adsorption isotherms are obtained, and the open-metal site contribution to the overall CH_4_ usable capacity is evaluated. It is found that sufficient ionic character is required, as exemplified by the strong CH_4_ affinities of 2,2′-bipyridine-CaCl_2_ and Mg, Ca-catecholate. In addition, it is found that the capacity of a single metal site depends not only on its affinity but also on its geometry, where trigonal or “bent” low-coordinate exposed sites can accommodate three or four methane molecules, as exemplified by Ca-decorated nitrilotriacetic acid. The effect of residual solvent molecules at the open-metal site is also explored, with some positive conclusions. Not only can residual solvent stabilize the open-metal site, surprisingly, solvent molecules do not necessarily reduce CH_4_ affinity, but can contribute to increased usable capacity by modifying adsorption interactions.

## Abstract 2

Open-metal sites are shown to significantly increase the CH_4_ storage capacity of porous materials. It is shown that the capacity is not determined solely by their CH_4_ affinity, but also by their geometry as well as by guest molecules.

## Graphical abstract

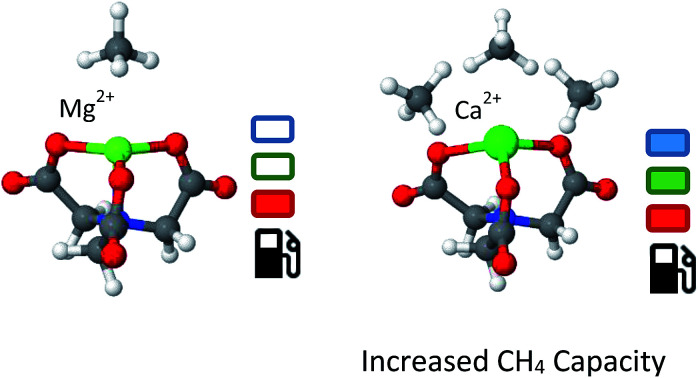

## Introduction

Natural gas (NG) is a naturally occurring mixture of hydrocarbons, consisting mostly of methane (CH_4_). NG is predominantly used for domestic, commercial and industrial applications such as heating, cooking and electric power production. NG is also used as an alternative fuel source for the transportation sector,^1,2^ and has several advantages over gasoline. Methane has the highest ratio of hydrogen to carbon of all fossil fuels and thus has the highest gravimetric energy density. Because of this, NG burns cleaner than gasoline and emits lower levels of greenhouse gases through its life cycle,^3^ although capture of waste CO_2_ at the source is a critical separation challenge in light of climate change considerations. Additionally, recent advances in horizontal drilling and hydraulic fracturing technologies are expected to increase NG production and sustain its low price.^4^

The main challenge in using NG as a fuel for transportation is that it has a much lower volumetric energy density at ambient conditions (0.04 MJ L^−1^) than gasoline (32.4 MJ L^−1^). Therefore, NG cannot be practically used for passenger vehicle applications in its standard state. Instead, NG is currently stored on-board the vehicle in either compressed (CNG) or liquefied (LNG) forms; however, these require expensive and bulky equipment for storage, compression and thermal isolation. A different approach to on-board storage involves having the NG stored in an adsorbed state inside a porous material with a high surface area. In the adsorbed state, the effective volume of the gas molecules is considerably smaller than in the gaseous state, which enables storage of larger quantities in lower volumes and at lower pressures. Over the last several decades, studies on adsorbed natural gas (ANG) have focused mostly on activated carbons;^5^ however, in recent years metal–organic frameworks (MOFs) have emerged as promising storage media.^6^

MOFs are a family of compounds consisting of metal ions or clusters coordinated to organic ligands (linkers), which form extended network structures.^7^ Due to their tunable pore sizes, MOFs have attracted attention for their potential use as gas-storage media. Furthermore, MOF composition and structure can be modified and tuned, as evidenced by their many other potential applications, including in catalysis^8,9^ and chemical separations.^6,10,11^

In 2012, the US Department of Energy (DOE) outlined the latest targets for ANG storage, which require a volumetric density of 263 v STP/v at maximum pressure of 35 bar, equivalent to the same density of CNG at 250 bar and 25 °C. For activated carbons, the most recent reports on volumetric CH_4_ capacities are in the range of 130–170 v STP/v, well below the DOE target.^12^ But even for MOFs, a recent study indicates there exists an upper bound for the usable capacity at 65 bar of approximately 200 v STP/v, also well below the DOE target.^13,14^ Known MOFs with the highest CH_4_ capacities, such as MOF-5,^15,16^ HKUST-1,^17^ UTSA-76a^18^ and MOF-519,^19^ are already at or close to that boundary. Evidently, to get closer to the DOE target, the attraction between CH_4_ and its host material should be increased beyond what is currently achievable by MOFs or activated carbons, for example by binding multiple CH_4_'s to a given site.

A promising strategy for augmenting the CH_4_ capacity of MOFs is by introduction of open-metal sites into their structure.^20–22^ Most open-metal sites have a significantly stronger interaction energy with CH_4_ than other adsorption sites on the pore surface of a MOF, and therefore, MOFs containing high concentrations of open-metal sites are expected to have higher CH_4_ capacities. It is important to note that the usable (also known as “deliverable”) CH_4_ capacity is more important than the total CH_4_ capacity for ANG applications. The usable capacity is defined as the difference between the amount of CH_4_ adsorbed at the maximum adsorption pressure, typically 35–65 bar, and the amount that is still adsorbed at the minimum desorption pressure. For ANG storage, the minimum desorption required for gas to flow from the fuel tank to the combustion engine depends on the specific requirements of each vehicle, but 5.8 bar is often assumed for initial comparisons between materials. The optimal adsorption enthalpy (Δ*H*_ads_) for maximizing usable CH_4_ capacity at ambient conditions has been shown to be about −17 kJ mol^−1^.^23^ Higher enthalpies result in over-adsorption of the CH_4_ at low pressures, while lower enthalpies result in under-adsorption at higher pressures. In addition to an optimal Δ*H*_ads_, sufficiently high densities of open-metal sites are needed to achieve high usable capacities.

Insertion of open-metal sites into MOFs can be realized by the post-synthetic modification (PSM) approach,^22,24,25^ whereby the MOF is first synthesized and then afterwards the open-metal site functionality is introduced. The separation of the synthesis process into two (or more) steps can be necessary, since the open-metal sites are chemically active and can prevent the formation of the MOF. While many MOFs have open-metal sites as part of their frameworks, the PSM approach is more widely applicable to any MOF that has metal chelating sites present on the organic linkers. Furthermore, there is an additional flexibility in potential types and composition of open-metal sites, such that open-metal sites with rationally tailored properties are possible. An important challenge related to the practical use of open-metal sites is the likely presence of solvent molecules: at the end of MOF fabrication, the open-metal sites are usually passivated by the presence of coordinated solvent molecules or charge-balancing anions. In order for the MOF to realize its full CH_4_ adsorption potential, these solvent molecules must be fully removed during activation.^26^ The activation of strongly interacting open-metal sites is expected to be challenging (or even impossible) due to the strong metal–solvent interaction, which can be on the order of −100 kJ mol^−1^, several times stronger than the expected interaction with CH_4_.

Although this introduction is focused on MOFs, the open-metal sites insertion approach, as well as the issues related to the presence of solvent, are completely general and are relevant to any other porous framework material, such as covalent–organic frameworks^27,28^ (COFs) and porous-aromatic frameworks^29,30^ (PAFs).

In this study, the adsorption of CH_4_ on several types of open-metal sites, both known and hypothetical, is explored. Also, the presence of solvent molecules, and their effect on CH_4_ adsorption, is studied in detail. By analyzing the nature of the underlying interactions, we attempt to provide an intellectual framework for understanding metal site-CH_4_ chemistry and provide guidelines for the design of effective NG storage materials. The selection of the studied metal-ions is mostly driven by practical considerations: a suitable ion should have an adequate adsorption energy, be lightweight, cheap and environmentally benign. In these respects, the alkaline-earth metals Mg and Ca seem more adequate than the first-row transition metals, since due to their strong ionic character, they are expected to have strong interactions with CH_4_, and are also earth-abundant and non-toxic. Additionally, due to their closed shell character, they are not expected to be involved in any strong covalent interactions with, or to activate, the C–H bonds of CH_4_.

The manuscript is organized as follows: the Model and computational details section reviews and discusses: (1) the cluster model, (2) the thermodynamic model for adsorption isotherms, (3) usable capacity, (4) energy decomposition analysis, and (5) computational details of the DFT calculations. The Results section is divided into several parts, each examining the CH_4_ adsorption on a specific type of system: (1) bare metal ions, (2) catecholate linker metal-sites, (3) bipyridine linker metal-sites, (4) nitrilotriacetic acid metal-sites, and (5) solvent effects in catecholate metal-sites. The Discussion and conclusion section is divided into (1) expected usable capacities and (2) conclusion.

## Model and computational details

### Cluster model

In this study, the CH_4_ interactions with open-metal sites are studied using a cluster model, which consists of a single adsorption site, made from a single metal ion complexed to a linker molecule (*i.e.*, a metalated linker) with one or more adsorbed CH_4_ molecules. MOFs are composed of well-defined ligand subunits that, to a reasonable approximation, maintain their individual precursor (pre-MOF) structures. Since the CH_4_ adsorption site has approximately the same geometry as in its pre-MOF molecular state, cluster models are able to provide valuable computational description of, and insights into, the interaction between CH_4_ and the adsorption site. Moreover, cluster models enable an accurate computational description of linker–metal–methane–solvent systems, utilizing higher level electronic structure theory methods than can be applied to periodic MOF models. For these reasons, cluster models have been successfully used in recent studies to model small molecule adsorption in MOFs.^9,31–36^ In spite of these successes, cluster models have yet to be extensively used and tested for prediction of adsorption isotherms and usable capacities, as described in below.

A cluster model is a good approximation of the MOF environment only in the vicinity of an open-metal site and its linker, as at larger distances other effects introduced by the MOF environment become significant. We therefore truncate our model after the first coordination/solvation shell of the metal. Other adsorption sites besides the open-metal sites, are also present in the MOF and contribute to the overall CH_4_ capacity. Especially at higher occupancy (high pressures), a CH_4_ molecule adsorbed on an open-metal site can interact with CH_4_ on other adsorption sites, or gaseous CH_4_, resulting in slightly increased adsorption energy. However, this study is focused on understanding open-metal site-CH_4_ interactions, which are only weakly dependent on the extended MOF environment, and therefore a cluster-model approach is appropriate. An estimate of the usable CH_4_ capacity, useful for qualitative comparisons between different open-metal sites, can be made on the basis of these calculations, and is described below.

### Thermodynamic model of adsorbed CH_4_

To connect between the cluster model calculations and experimental observables, such as usable capacity, a simple equilibrium model for estimating metalated linker site occupancy and the associated site adsorption isotherms will be used. The model assumes that each metalated linker (*L*) can adsorb up to a maximum of *S* methane molecules (the first solvation shell). A metalated linker with *i* adsorbed molecules is denoted by *L*_*i*_. Methane cannot adsorb on the linker to form *L*_*i*_ unless *L*_*i*−1_ already exists. Thus a sequence of binding equilibria exists:1
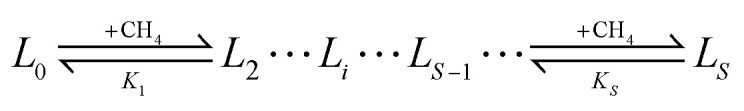


The associated equilibrium constants, *K*_*i*_, are straightforwardly given by:2
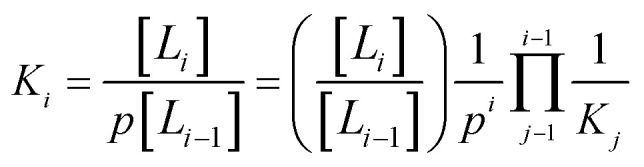
here, [*L*_*i*_] is the fraction of metalated linkers with *i* adsorbed gas molecules, and *p* is the pressure of the gas. The key observable is the average number of adsorbed molecules at each metalated linker, *θ*(*p*), which can be evaluated in terms of *p* and *K*_*i*_ using eqn (1) as:3
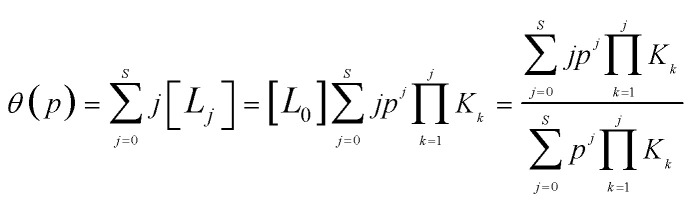
the value of *θ*(*p*) can swing between 0 (no adsorbed molecules) and *S* (maximum number of adsorbed molecules). If the linker can only adsorb a single molecule (*i.e.*, *S* = 1), eqn (3) reduces to the well-known Langmuir equation, *θ*(*p*) = *pK*_1_/(1 + *mK*_1_).

The equilibrium constants *K*_*i*_ are evaluated using the standard expression, *K*_*i*_ = exp(−Δ*G*_*i*_/*RT*), where Δ*G*_*i*_ is the differential free energy of adsorption of the *i*^th^ CH_4_ molecule. The evaluation of Δ*G*_*i*_ requires both the adsorption entropy, Δ*S*_*i*_, and the adsorption enthalpy, Δ*H*_*i*_. The nuclear motion contributions are usually evaluated in quantum chemical (QC) calculations by assuming that the adsorbent–adsorbate complex is a collection of harmonic oscillators with only vibrational degrees of freedom. When the adsorbing molecule interacts weakly with the adsorbent, the adsorbed molecule retains approximately 2/3 of its translational and rotational freedom,^37^ however, the harmonic approximation, commonly used in QM calculation of calculation of the thermodynamic partition function, cannot take into account that certain lower-frequency motions, common for inter-molecular physical interaction, should be treated as hindered internal rotations or translations, rather than as vibrations.^38^ Since the translational degrees of freedom have much higher entropy than the vibrational, and vibrations have much higher internal energy than translations, the treatment of motions as vibrations, rather than hindered translations, results in an underestimation of the entropy and overestimation of the (positive) internal energy of the complex.

Since rigorous evaluation of Δ*G*_*i*_ is unfeasible, we calculate Δ*U*_*i*_*via* the difference of electronic energies from QC calculations and adopt simple, though reasonable, approximations for the remaining contributions. As discussed in the ESI,† Δ*S*_*i*_ is assumed to be independent of *i*, and set to the value of −9.5*R* which represent an intermediate value measured for materials for adsorptive storage applications.^23,39^ The value of Δ*H*_*i*_ is assumed to be given by Δ*U*_*i*_ + Δ*U*_vib_ − *RT*, based on the *pV* contribution, and the change in vibrational energy. Upon adsorption of a CH_4_ molecule, a weak “physical” bond (50 to 150 cm^−1^) is formed by restriction of the CH_4_ movement through conversion of one translational degree of freedom into one “bonding” vibration, whose contribution Δ*U*_vib_ is, to a good approximation, 2.5 kJ mol^−1^ at ambient conditions.

We assume that contributions to the MOF usable capacity from second layer coverage is of minor importance at the pressure range considered here of less than 65 bar, as we truncate adsorption upon closing of the first solvent shell (a characteristic number, *S* molecules for a given binding site). Furthermore, this model does not attempt to simulate adsorption isotherms for any specific MOF, as this requires larger models that are individually tailored for specific MOFs. Rather, the model enables qualitative comparison of the usable capacity associated with different binding site designs, in a given framework. As described below, we will choose existing pre-metalated frameworks for this purpose.

### Usable capacity

An internal combustion engine in a passenger vehicle operates across a range of CH_4_ pressures. When the ANG fuel tank is full, the pressure is maximal (*p*_max_).

As the NG is desorbed and flows into the engine, the pressure gradually drops down to a minimal pressure, which is needed by the engine to operate (*p*_min_). In this work, *p*_max_ is chosen as either 35 bar, which is the maximal pressure that is obtained using a single-step compressor, or *p*_max_ = 65 bar, which is also used in the literature. The value of *p*_min_ is taken to be 5.8 bar.

**Fig. 1:**
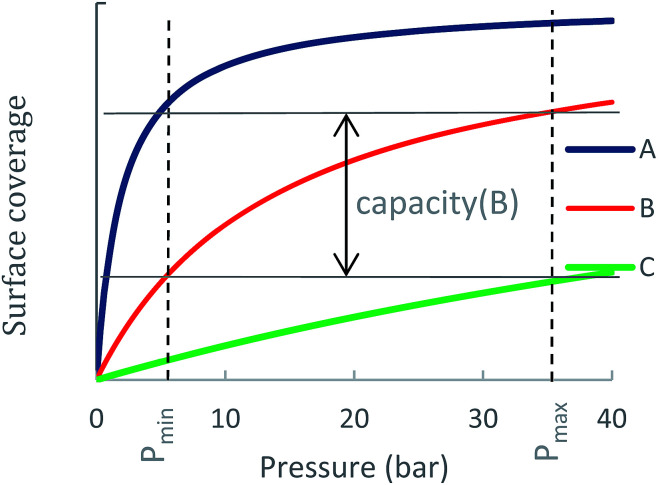
Usable capacity of three different ANG materials A, B and C, where Δ*H*_ads_(A) > Δ*H*_ads_ (B) > Δ*H*_ads_ (C). The usable capacity is proportional to the difference between surface coverage at maximal and minimal pressures. The usable capacity of B is larger than A and C since A is over-adsorbing and C is under-adsorbing.

An ideal storage material (or a metal-site) should be able to swing between high surface coverage at *p*_max_ and low surface coverage at *p*_min_. To illustrate this consideration, Fig. 1 shows adsorption curves of three different ANG materials A, B and C, each having a different NG adsorption enthalpy, Δ*H*_ads_(A) > Δ*H*_ads_(B) > Δ*H*_ads_(C). Material A is a strong NG adsorbent: at *p*_max_ its surface is completely covered, *i.e.*, a large amount of CH_4_ is adsorbed. However, at *p*_min_ the coverage of A remains high such that only a small amount of CH_4_ is released to the engine and the usable capacity is sub-optimal. Material C is a weak CH_4_ adsorbent: at *p*_max_ peak coverage is not reached, and C contains a relatively small amount of CH_4_ and is therefore not able to store NG optimally. Material B has an optimal Δ*H*_ads_ for CH_4_ storage at ambient conditions: although it does not reach full coverage at *p*_max_, its ability to sufficiently release CH_4_ at *p*_min_ gives B a better usable capacity than A and C. Notably, for cases such as A, where usable capacity is limited by over-attraction for CH_4_, heating could be applied to the storage tank at lower pressures to facilitate the release of strongly adsorbed CH_4_ molecules, thereby increasing usable capacity.

To distinguish the usable capacity of the MOF from the contribution of the individual metal-site that are calculated, we define the metal-site's usable occupancy as the difference between the number of adsorbed molecules on a metal-site at *p*_max_ and at *p*_min_:Δ*θ*_uo_ = *θ*_*p*_max__ − *θ*_*p*_min__Δ*θ*_uo_ is an intrinsic property of the site itself, regardless of the site's concentration or the MOF environment, which represents the number of CH_4_ molecules that are desorbed from the metal-site as pressure swings from high to low. Since the metal site can be occupied by several CH_4_ molecules, the term “site occupancy” is used to describe adsorption on the metal sites, rather than term “surface coverage” which is related to the macroscopic property of the material.

To increase the readability of the paper, the expected usable site occupancy of the open-metal sites is compared to that of the clusters in the parent MOFs (see Tables 3, 5, 6 and 9). This is done by rewriting the Langmuir equation, to obtain *θ*_cluster_, cluster occupancy values that are comparable to *θ*_oms_, the occupancy of the open-metal sites. Further details are found in the ESI.† The ESI† also contains a section which evaluates the methodology used here for estimation of usable capacities, to experimental gas-measurement results for the well-characterised MOF-5 system. Quantitative agreement is obtained between the model and experiment.

We emphasize that the interactions reported are the computationally calculated energies, and not the predicted experimentally measurable enthalpies. Conversely, the predicted adsorption isotherms and derived site occupancies, do incorporate certain data and assumptions regarding the thermodynamic nature of the systems, and therefore can in principle be compared to experiment, subject to limitations in modeling discussed already.

### Energy decomposition analysis

The physisorption of CH_4_ is essentially non-chemical because it does not involve the formation or breakage of chemical bonds. Therefore, standard wave-function analysis concepts, such as partial charges and bond orders, cannot provide an adequate picture of the key contributions to physisorption. Instead, we employ “Energy Decomposition Analysis” (EDA),^40,41^ as implemented in the Q-Chem quantum chemistry package,^42^ which decomposes the interaction energy of two or more molecules, into three contributions: (1) frozen (FRZ), (2) polarization (POL) and (3) charge transfer (CT).

4 *E*_Interaction_ = *E*_FRZ_ + *E*_POL_ + *E*_CT_

the FRZ term corresponds to the energy change due to interactions that are not related to a change in the Kohn–Sham orbitals of the interacting molecule, *i.e.*, electrostatic interactions due to permanent multipoles, dispersion and steric repulsion. The POL term corresponds to the energy change due to the polarization of the density of each molecule, while constrained to remain localized on the molecule. The CT term corresponds to energy change due to the flow of charge between the polarized molecules.

In more detail, the EDA uses the “SCF-MI”^43,44^ approach for obtaining an “Absolutely Localized Molecular Orbital” (ALMO) wave-function *Ψ*_ALMO_. The ALMOs prohibit CT by fragment-blocking the MO coefficient matrix. The FRZ term is the energy required to bring infinitely separated molecules into the complex geometry, using the frozen MOs of the fragments: 
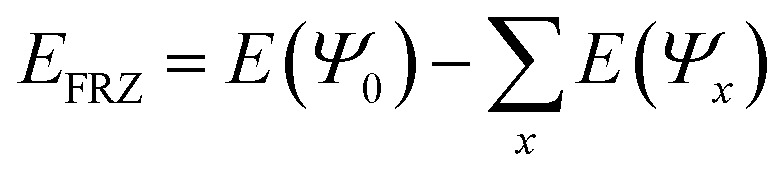
. The POL term is evaluated as the difference between the energy of the optimized ALMO wave-function and the frozen wave-function: *E*_POL_ = *E*(*Ψ*_ALMO_) − *E*(*Ψ*_0_). The CT term is evaluated as the energy difference between the energy of the fully converged SCF wave-function of the complex and the CT-excluded ALMO energy: *E*_CT_ = *E*(*Ψ*_SCF_) − *E*(*Ψ*_ALMO_). The positive energy related to the geometric distortion of the molecule in its complex geometry with respect to its isolated geometry is called the “geometric distortion” (GD) energy. For the sake of readability, only the parts of the EDA which are essential for the discussion are shown while the full EDA is found in the ESI.†

This work also employs complementary occupied-virtual orbital pairs (COVPs)^45^ for visualization of the intermolecular CT interactions. The COVPs are a chemical representation of inter-molecular CT in simple terms of donor–acceptor orbital pairs that provide a compact representation of the most significant donor–acceptor orbital interactions.

### Computational details

All calculations are carried out using the meta-GGA B97M-V density functional,^46^ which utilizes the VV10 non-local correlation functional for its treatment of dispersion interactions.^47^ The VV10 term relies only on electronic densities to compute the dispersion interactions, without any predetermined atom-specific parameters. Therefore, this dispersion treatment is more transferrable compared to other dispersion treatment approaches, and can be applied successfully to a wider range of systems, not specifically included in benchmark datasets. A recent benchmark by Herbert and coworkers on intermolecular interactions involving ions demonstrated that B97M-V performs very well for anion-neutral dimers, cation-neutral dimers and ion pairs.^48^

The structures are initially optimized using the 6-31g* basis set and validated to be a minimum on the energy potential surface using a standard frequency analysis, validating that the correct conformation of the structures is obtained. These geometries are then further optimized using the triple-zeta def2-tzvp basis set^49^ which are then used, along with the larger quadruple-zeta def2-qzvp basis set,^50^ for evaluation of interaction energies and EDA analysis. The reported adsorption energies and charge transfer interactions are counterpoise corrected for basis set superposition error. The insensitivity of the geometry optimizations to additional diffuse functions was tested on the CH_4_ dimer, cat-Mg and cat-Ca, using the diffuse def2-tzvpd basis, which resulted in similar structures and interaction.

Due to the shallow nature of the potential energy surface of the weakly interacting molecules, there is an inherent challenge in the exact identification of the global minimum structures. This is particularly true for partially solvated structures. Thus, two different geometry optimizations from different initial guesses, may converge into geometrically similar structures but having slightly different energies, of about ±1 kJ mol^−1^, which can be considered as an additional uncertainty of these calculations.

## Results

### Methane clusters formed by metallic ions

We start the study of CH_4_ adsorption by looking at the simplest models of CH_4_ molecules interacting with a bare ion, forming a solvation shell. Due to the strict electrostatic nature of the alkali or alkaline-earth metal ions, these clusters can provide useful insights into the interaction of the CH_4_ with the strong electrostatic fields induced by the ions. It is emphasized, that in spite of the simplicity of the models, this is not a purely theoretical case-study, as doping of porous materials with lithium ions is a well-recognized strategy for enhancing the adsorption of CH_4_, as well as other species.^51–53^

**Fig. 2:**
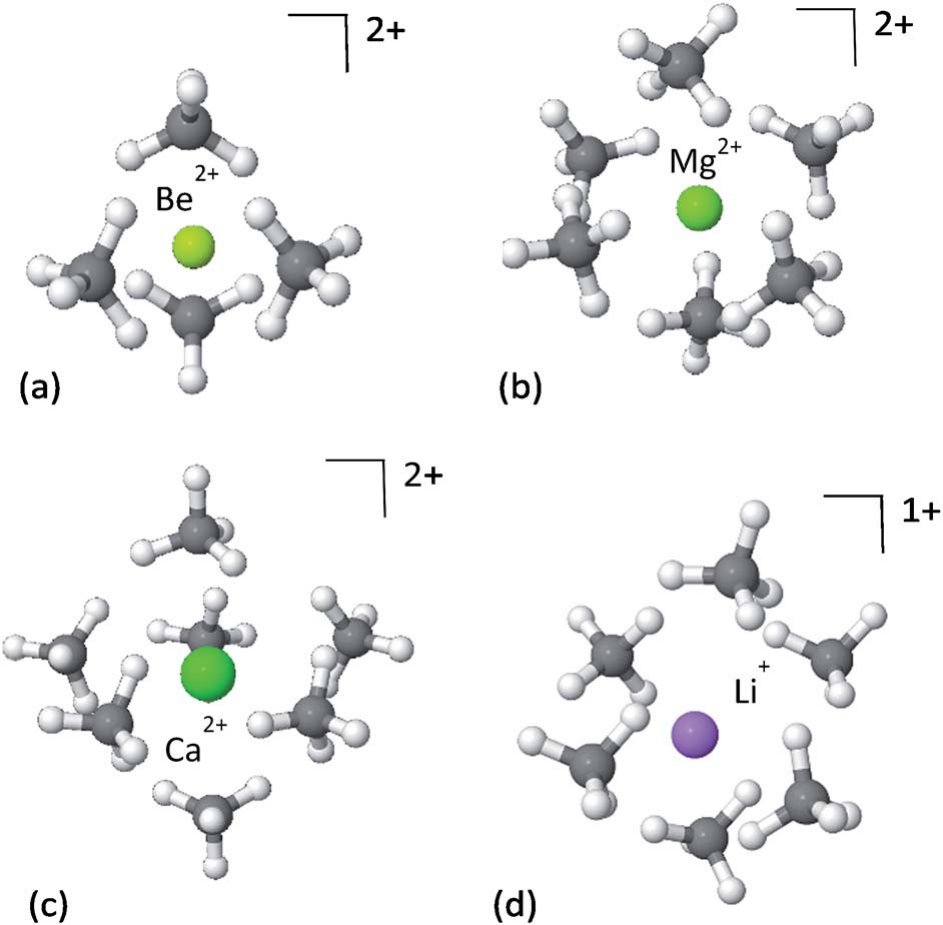
First solvation shell of methane clusters formed around bare ions: (a) Be^2+^–4CH_4_, (b) Mg^2+^–6CH_4_, (c) Ca^2+^–7CH_4_ and (d) Li^+^–6CH_4_. Although the Li^+^ complex is considerably less charged than the Mg^2+^ complex, they are able to coordinate the same number of CH_4_ molecules due to their similar radii. However, their adsorption energies are different.

The panels in Fig. 2 show the first solvation shell formed by CH_4_ molecules around Be^2+^, Mg^2+^, Ca^2+^ and Li^+^ ions, and the corresponding EDA is shown in Table 1. Since the CH_4_⋯CH_4_ interactions are relatively weak, on the order of −2 kJ mol^−1^, the dominant interactions driving the formation of the clusters are the cation–CH_4_ interactions.

**Table 1:** Energy decomposition analysis of clusters of CH_4_, formed around Be^2+^, Mg^2+^, Ca^2+^ and Li^+^. The results are given in kJ mol^−1^ and normalized per CH_4_ molecule. The clusters correspond to a complete first solvent shell

	Be^2+^(CH_4_)_4_	Mg^2+^(CH_4_)_6_	Ca^2+^(CH_4_)_7_	Li^+^(CH_4_)_6_
FRZ	11.0	0.0	4.2	−0.2
POL	−234.2	−103.6	−64.8	−24.7
CT	−36.2	−11.2	−15.1	−5.1
Total	−259.3	−114.8	−75.7	−30.0

The strongest attractive force in these clusters is polarization of the CH_4_, induced by the electrostatic fields of the cations. This is unsurprising given the chemical stability of methane, which means CT will be relatively small. The strength of the polarization is correlated with the ionic radius, such that the smaller the radius, the stronger the polarization. The ionic radii of Be^2+^, Mg^2+^ and Ca^2+^ are 59, 86 and 114 pm, respectively,^54^ as compared to the corresponding polarization energies of −234.2, −103.6 and −64.8 kJ mol^−1^. Smaller radii enable closer proximity of CH_4_ to the metal ion, where the electrostatic field of the ion is much stronger, and thus the induced polarization of the methane is also stronger.

Perhaps surprisingly, in spite of its smaller charge, Li^+^ coordinates the same number of CH_4_ molecules as Mg^2+^. This implies that the maximal coordination number of the ion is better correlated to ionic radius than charge, and reflects methane packing effects. This is supported by the close similarity between the ionic radius of lithium (90 pm) and that of magnesium (86 pm). However, because of its smaller charge the electrostatic fields near Li^+^ are weaker than near Mg^2+^, and the resulting polarization in the CH_4_ molecules is considerably less.

**Fig. 3:**
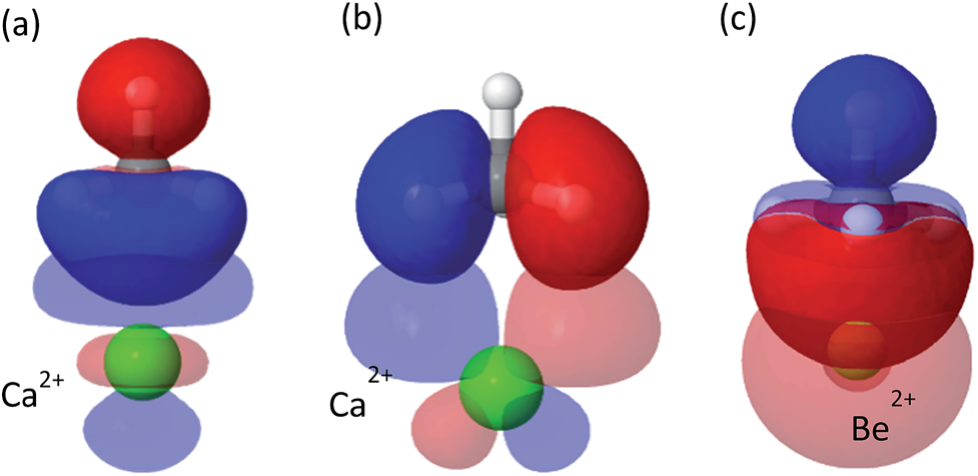
Significant donor–acceptor pairs for the charge-transfer interaction from CH_4_ to Ca^2+^ and Be^2+^. Panel (a) shows a σ-symmetry donation from C–H(CH_4_) to Ca^2+^ with associated energy lowering of Δ*E* = −4.3 kJ mol^−1^ and panel (b) shows π-symmetry donation from C–H(CH_4_) to Ca^2+^ with energy lowering of Δ*E* = −2.6 kJ mol^−1^. Panel (c) shows CT from CH_4_ to Be^2+^ where the C–H bonds donate into the 2s orbital.

Although less important than polarization, charge transfer has a significant contribution to the interaction energy of the clusters. Its absolute measure is close for the Mg^2+^ and Ca^2+^ clusters (−11.3 and −15.1 kJ mol^−1^), however, it is relatively more important for Ca^2+^ where it accounts for 20% of the attractive energy. COVP analysis (Fig. 3) indicates that charge is transferred mostly from the C–H bonds of the CH_4_ into high-valence orbitals on the metal. These CT interactions are closely related to the covalent (CT) interactions between C–H groups and transition metals, also known as “agostic interactions” in the context of the chemistry of C–H bond.^55^ However, for the cases discussed here, the vacant d-orbitals of the main group ions are too diffuse and high in energy for strong C–H(CH_4_) to d(M) orbital interactions. Therefore, the ion mostly polarizes the CH_4_ without activating the C–H bonds—a desirable property for storage applications. As could be expected,^56^ CT is considerably stronger for Be^2+^, which could be related to the fact that the valence orbitals of Be^2+^ are the 2s and 2p shells,^57^ and not d orbitals (see Fig. 3c). The small radius and strong polarization of Be^2+^ also contribute to physical proximity, which increases CT. It is also interesting to note, that CT per CH_4_ is similar for Mg^2+^ and Ca^2+^, which implies that CT is not necessarily proportional to the polarization induced by the ion.

Under realistic conditions, the metal ion is never completely free and would be either solvated with its electrostatic field shielded by the solvent or be in close proximity, or coordinated, to some balancing counter-ion or ligand. In other words, under realistic conditions the metal ion would have a reduced electrostatic field and carry a reduced charge – both of which are expected to weaken the interaction with CH_4_. Additionally, there are steric constraints associated with direct coordination.

As an illustration of the consequences of these effects, the maximal coordination number of the Ca^2+^ ion is reduced to 6CH_4_ molecules, instead of 7, as will be shown below.

### The catecholate (cat) linker

**Fig. 4:**
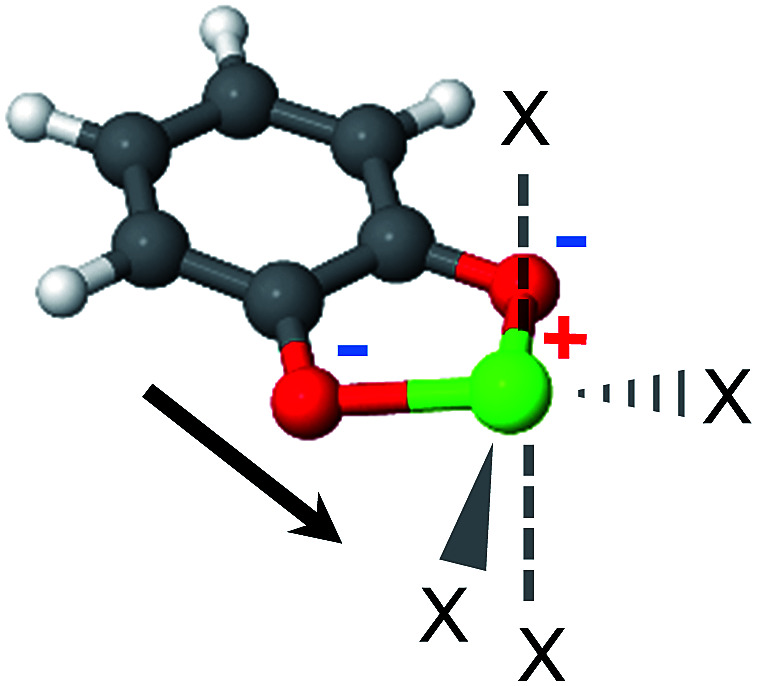
The catechol-Mg metalated linker. The electrostatic dipole moment (arrow) is formed by the two negatively charged O atoms and the positive metal. ‘X’ marks potential CH_4_ adsorption sites. The positive and negative formal charges are marked by red and blue colors, respectively.

The MOF linkers discussed first are based on the catecholate (cat) ligand, which is a commonly used chelating agent throughout coordination chemistry. A key feature of the catecholate-(Mg, Ca) linker, as shown in Fig. 4, is that the arrangement of the electrostatic charges around the metal ion results in a strong dipole moment. Moreover, the catecholate is a bidentate ligand, such that the metal ion coordination sphere still has 4 vacant coordination sites. Metalated catecholate linkers have been previously studied in the context of H_2_ storage^58–60^ and shown to be capable of strong physical interactions with H_2_.

MOFs containing catechol ligands were recently synthesized and metalated by Fe^3+^, Cr^3+^ and Pd^2+^.^61–63^ The specific metalations discussed here have yet to be attained, however, there is experimental evidence^64^ for the preparation of catecholate complexes with Mg^2+^, and other studies, focused on the role of melamine in biological Ca^2+^ regulation, indicate that both Ca^2+^ and Mg^2+^ form complexes with catechol even in aqueous solutions.^65–69^

**Fig. 5:**
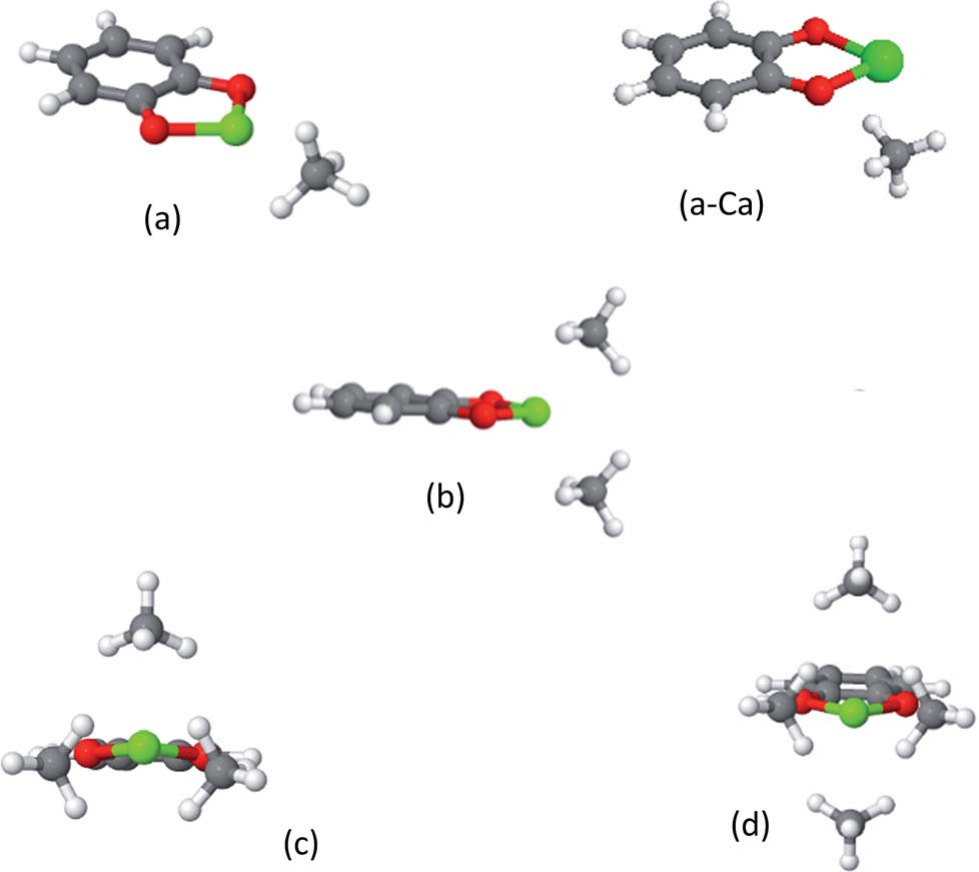
Clusters of CH_4_ on cat-Mg. Cat-Ca forms similar structures, except for the case of the 1CH_4_ molecule, which is shown in panel (a-Ca). The strong electrostatic response of the CH_4_ in (a) can be deduced from its alignment along the dipole moment of cat-Mg. As shown in panel (b), two CH_4_ molecules complete tetrahedral coordination around Mg or Ca, while three and four CH_4_ molecules partially complete or complete an octahedral coordination environment, as shown in panels (c) and (d).

The optimized structures of the clusters of CH_4_ with catechol-(Mg, Ca) complexes are shown in Fig. 5, and the corresponding EDA is provided in Table 2. It can be seen that cat-Mg and cat-Ca share similar structural characteristics for their interaction with methane molecules. In both cases, CH_4_ gradually fills the coordination sphere of the ion, forming a tetrahedral structure for 2CH_4_ and an octahedral structure for 4CH_4_. However, the interaction energy change (Δ*E*_ads_) for the addition of each CH_4_ molecule shows different trends: it is decreasing for cat-Mg while only slightly decreasing for cat-Ca.

**Table 2:** Polarization and adsorption energy of CH_4_@catechol-(Mg/Ca) clusters. Units are given in kJ mol^−1^

*N* ^th^ CH_4_	Cat-Mg		Cat-Ca	
	ΔPOL	Δ*E*_ads_	ΔPOL	Δ*E*_ads_
1	−49.9	−40.7	−19.9	−26.7
2	−33.6	−27.0	−17.6	−24.7
3	−17.9	−14.6	−16.0	−21.7
4	−1.0	−17.1	−16.4	−22.2

The EDA can provide useful insights into this behaviour. For both cat-Mg and cat-Ca the dominant attractive interaction is the polarization, *i.e.*, the electrostatic response of the CH_4_ to the electrostatic fields of the ion. The polarization interaction for cat-Mg is expected to be larger than for cat-Ca, due to the smaller size of the Mg^2+^ ion—as was demonstrated above for the Mg^2+^ and Ca^2+^ clusters. Indeed, the polarization energy for the first coordinated CH_4_ molecule is much stronger for cat-Mg than for cat-Ca (−49.9 *vs.* −19.9 kJ mol^−1^), which also results in them having different geometries. Since Mg is small with respect to methane, it can be efficiently shielded by CH_4_, such that the electrostatic field of the Mg^2+^ ion is decreased significantly with the addition of each CH_4_.

Indeed, the fourth coordinated CH_4_ is polarized only by −1.0 kJ mol^−1^*vs.* −49.9 kJ mol^−1^ for the first. The radius of Ca^2+^ is larger than Mg^2+^; and therefore, CH_4_ does not as efficiently shield its electrostatic field.

The importance of the electrostatic interaction between the CH_4_ and the static charges of the cat-Ca is demonstrated by examining the structure of the cat-Ca cluster with a single CH_4_ molecule in Fig. 5(a-Ca): the two positive H atoms in CH_4_ are aligned under the two negative O atoms in cat-Ca, while the negative C atom in CH_4_ is aligned under the positive Ca^2+^ ion.

**Fig. 6:**
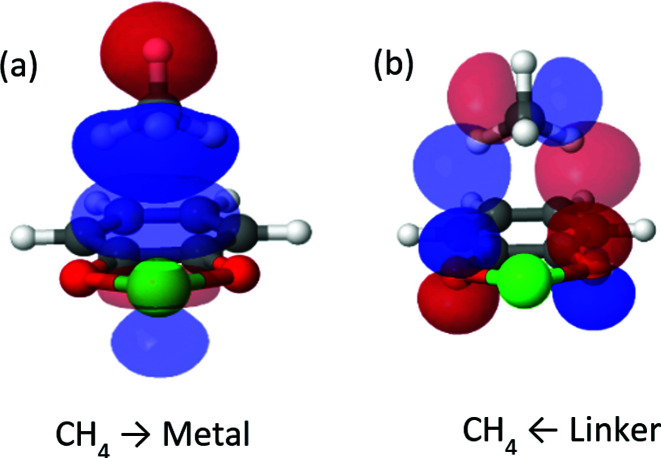
Most important complementary occupied-virtual pair orbitals in cat-Ca. Panel (a) shows forward donation from methane to metal, panel (b) shows backward donation.

CT interactions (see Fig. 6) are approximately the same for both cat-Mg and cat-Ca, on the order of approximately −4 to −5 kJ mol^−1^. Charge is transferred from the C–H bonds of CH_4_ into high valence orbitals on the metal, and charge is transferred back from π orbitals of the oxygens to vacant anti-bonding C–H* orbitals in CH_4_. Both backward and forward contributions are of approximately similar importance. Compared to the clusters studied above, the CT interactions are considerably weaker. Due to the presence of the coordinating catecholate linker: the acceptor orbitals of the metal ion are now already partly occupied prior to methane coordination.

#### Adsorption isotherms and MOF usable capacity

**Fig. 7:**
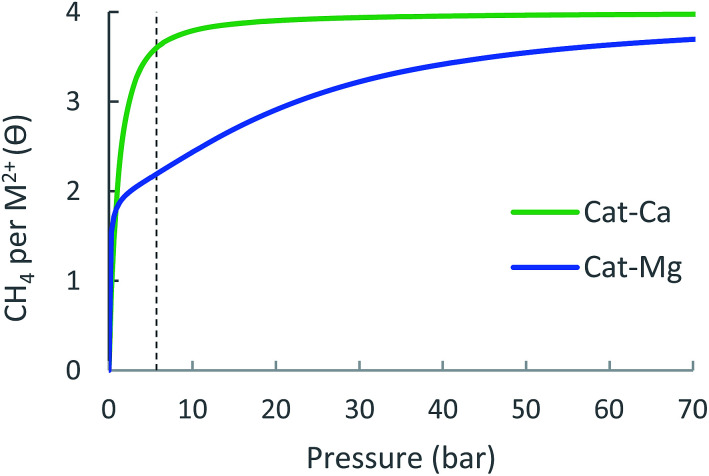
Expected metal-site adsorption isotherms for the cat-(Mg, Ca) at 298.15 K.

The adsorption isotherm of the cat-Ca and cat-Mg linkers are shown in Fig. 7 and details of the expected site occupancy are found in Table 3. Due the strong attractive forces of CH_4_ to the linker, the isotherms for both cat-Mg and cat-Ca are characterized by a fast increase in occupancy at low pressures, such that at 5.8 bar, cat-Mg sites are occupied by slightly more than two CH_4_ molecules and cat-Ca is occupied with about 3.5 molecules. In fact, the adsorption of the first CH_4_ to cat-Mg is so strong that its removal is probably not possible under standard conditions, as also evident from the very sharp increase in the occupation of cat-Mg at negligible pressure. Due to its over-attraction and quick saturation at low pressures, the cat-Ca site is able to achieve only a small usable occupancy.

**Table 3:** Usable metal site occupancy for cat-(Mg, Ca) at 298.15 K. Experimental data for MOF-5 is given for comparison

	Cat-Mg	Cat-Ca	MOF-5
Δ*θ*_uo_ (35 bar)	1.13	0.34	1.6
Δ*θ*_uo_ (65 bar)	1.47	0.37	2.6

For cat-Mg, however, the third and fourth CH_4_ molecules are adsorbed with weaker energies of −14.6 and −17.1 kJ mol^−1^, which are suitable for methane storage and thus contribute significantly to the usable site occupancy. Thus, at 35 bar the usable site occupancy of cat-Mg is 1.13, more than three times that of cat-Ca (0.34). We note that if a larger value for Δ*S*_ads_ is used, the usable site occupancy of cat-Ca increases, due to increased attenuation of its relatively strong interactions.

The ability of the metalated cat linkers to adsorb a large number of CH_4_ molecules does not come without challenges: due to their strong partial charge and strong dipole moment, the exposed metal ions could be attracted to negatively charged areas in the MOF framework, thus destabilizing it towards collapse of the porous structure. Also, solvent molecules present during synthesis could be difficult to remove during activation and block the CH_4_ adsorption sites. Later, we will return to the question of how tightly bound residual solvent molecules at the open-metal site may impact usable occupancy.

### The 2,2′-bipyridine (bpy) linker

**Fig. 8:**
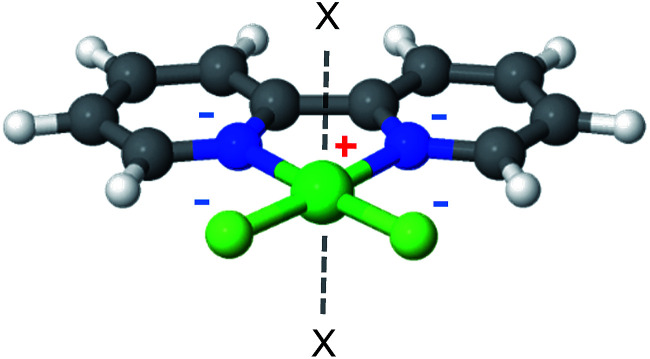
2,2′-Bipyridine metalated linker. ‘X’ marks potential CH_4_ adsorption sites.

2,2′-Bipyridine (bpy) is one of the most widely used ligands in coordination chemistry due to its strong affinity for metals. It is also commonly used as a MOF linking element, and there are several reports in the literature of MOFs containing metalated bpy units.^21,70–72^Fig. 8 shows a metalated bpy linker with two accompanying chloride anions for overall electrical neutrality. Assuming a square planar exposed metal site of this type, one expects two potential CH_4_ adsorption sites at the axial positions. Since the metal ion is coordinated by four negatively charged centers, which potentially reduce the strong-polarizing electrostatic fields, the adsorption mechanism of CH_4_ to the metal site is expected to be mostly frozen-electrostatic with the attraction of CH_4_ to the negatively charged counterions and positive metal. The most common metalation of bpy to date, as reported in three papers,^21,71,72^ is by PdCl_2_ and PtCl_2_, which results in a square planar complex of the type shown in Fig. 8. However, these complexes show only a weak interaction with CH_4_, exhibiting a binding energy of about 10 kJ mol^−1^, which is insufficient for CH_4_ storage. Here, we study the bpy-CuCl_2_, bpy-ZnCl_2_ and bpy-CaCl_2_ complexes and analyze their CH_4_ adsorption properties.

While MOF metalation by bpy-CuCl_2_ was recently demonstrated,^73^ there is also experimental evidence from both solution^74,75^ and the gas-phase^76^ for complexation of Ca^2+^ and Zn^2+^ with bpy. We note, that for practical purposes using larger pyridyl ligands, such as 1,10-phenanthroline would result in stronger metal-linker binding,^75,76^ and is another potential future synthetic target.

**Fig. 9:**
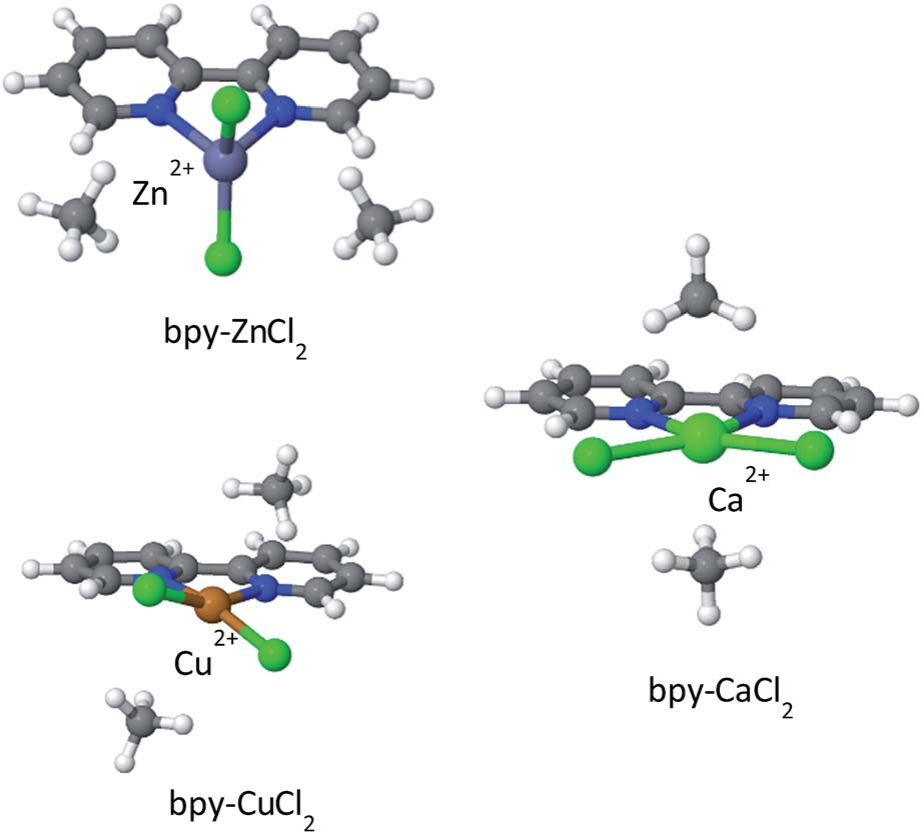
CH_4_ adsorbed on metalated bpy linkers. In the absence of adsorbed CH_4_ molecules, the structure of bpy-CaCl_2_ is close to that shown for bpy-CuCl_2_.

The optimized structures of the complexes are shown in Fig. 9; bpy-ZnCl_2_ is tetrahedral, bpy-CuCl_2_ and bpy-CaCl_2_ are predicted to be moderately distorted square planar complexes. Due to the larger size of the Ca^2+^ cation in bpy-CaCl_2_, it is less efficiently coupled (coordinated) to the bpy ligand, and therefore maintains some of its pre-coordinated ionic nature. The resulting geometry of the bpy-CaCl_2_ complex is intermediate between a fully coordinated tetrahedral structure, dominated by covalent interactions, and a planar structure dominated by the ionic interactions between the F^−^ counterions and the positive H of the bpy. When two CH_4_ molecules are adsorbed on the “naked” complex (not shown in the figure), the electrostatic interactions between the positive Ca^2+^ ion and the negative nitrogen atoms are screened, shifting the equilibrium towards the planar structure and reducing the “twisting” of the Cl^−^ counterions and shifting towards a planar position.

Similarly, bpy-CuCl_2_ which is also non-planar, is expected to have some small ionic contribution to its character.

Overall binding energies for one and two CH_4_ molecules, as well as the EDA, are shown in Table 4. The EDA indicates that bpy-CuCl_2_ and bpy-ZnCl_2_ have similar characteristics: both ΔCT and ΔPOL are on the order of −2.5 to −4 kJ mol^−1^, however, the frozen-electrostatic interactions are stronger for bpy-CuCl_2_ than for bpy-ZnCl_2_ by −3.2 kJ mol^−1^ for the first adsorbed CH_4_ molecule. The weaker frozen-electrostatic interaction for bpy-ZnCl_2_ is explained by the tetrahedral geometry of the Zn ion and thus being shielded, both electrostatically and sterically, by negative charges—the positive ion is therefore not available for attracting the negative carbon atom of CH_4_. Moreover, the ionic character of bpy-CuCl_2_, increases the frozen-electrostatic attraction forces for CH_4_. Increased ionic character is even more pronounced in bpy-CaCl_2_ and therefore the adsorption of CH_4_ to bpy-CaCl_2_ is much stronger than to bpy-CuCl_2_ by about −6.9 kJ mol^−1^ and is characterized by elevated charge-transfer and polarization interactions, due to the partially exposed Ca^2+^ ion. The frozen interactions for bpy-CaCl_2_ are relatively small, due to increased (positive) steric repulsion.

**Table 4:** EDA of bpy linker metalated with CuCl_2_, ZnCl_2_ and CaCl_2_. All values are in kJ mol^−1^

*N*th CH_4_	bpy-CuCl_2_				bpy-ZnCl_2_				bpy-CaCl_2_			
	ΔFRZ	ΔPOL	ΔCT	Δ*E*_ads_	ΔFRZ	ΔPOL	ΔCT	Δ*E*_ads_	ΔFRZ	ΔPOL	ΔCT	Δ*E*_ads_
1	−7.1	−2.8	−3.2	−12.9	−3.9	−2.6	−3.8	−10.1	−1.2	−11.8	−9.5	−19.8
2	−4.3	−4.1	−3.7	−10.2	−3.9	−2.5	−3.9	−10.1	−3.9	−11.4	−9.5	−21.3

We note that the interaction with CH_4_ could be increased, by about −1 kJ mol^−1^, by using F^−^ counter-ions that are more electronegative than Cl^−^.

#### Adsorption isotherms and usable site occupancy

**Fig. 10:**
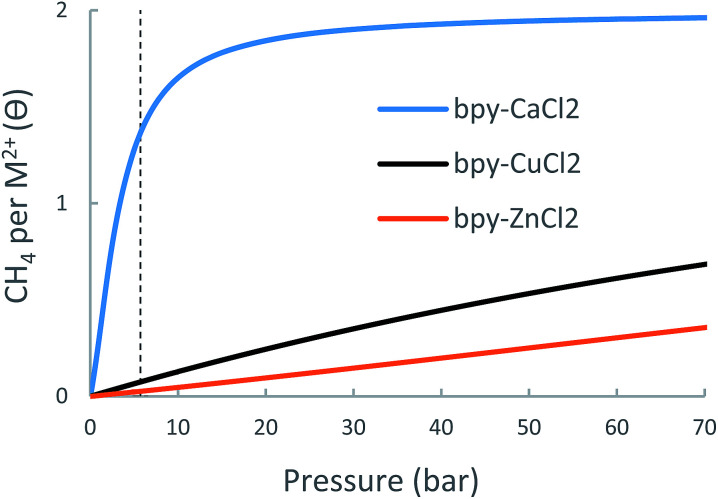
Calculated metal-site adsorption isotherms for the bpy-(CuCl_2_, ZnCl_2_, CaCl_2_) at 298.15 K.

The adsorption isotherms of the bpy-(CuCl_2_, ZnCl_2_, CaCl_2_) linkers are shown in Fig. 10 and details of the expected usable site-occupancy is found in Table 5. Due to the strong attraction of the first and second CH_4_ of −19.8 and −21.4 kJ mol^−1^ respectively, bpy-CaCl_2_ begins to saturate at relatively low pressures, such that at 5.8 bar it is already occupied by a more than one CH_4_ molecule, hence its usable site-occupancy is limited by over-biding. bpy-CuCl_2_ and bpy-ZnCl_2_, however, have lower interaction energies with CH_4_, such that even in 65 bar they are not occupied by more than a single CH_4_. Although relatively weak, the adsorption energy of the first CH_4_ in bpy-CuCl_2_ of −12.8 kJ mol^−1^ results in non-negligible usable site occupancy at higher pressures of 0.57 at 65 bar.

**Table 5:** Predicted metal-site usable occupancy for bpy-(CuCl_2_, ZnCl_2_, CaCl_2_) 298.15 K. Experimental data for UiO-67-bpy is given for comparison

	bpy-CuCl_2_	bpy-ZnCl_2_	bpy-CaCl_2_	UiO-67-bpy
Δ*θ*_uo_ (35 bar)	0.32	0.15	0.54	2.4
Δ*θ*_uo_ (65 bar)	0.57	0.30	0.58	3.4

While CH_4_ storage bpy-CaCl_2_ is non-optimal due to over-attraction and bpy-CuCl_2_ and bpy-ZnCl_2_ suffer from under-attraction, it is very possible that other forms of bpy-metalation would result in intermediate-strength interactions and better CH_4_ storage performance.

### The NTA ligand

To date, post-synthetic metalation of both the 2,2′-bipyridine and catechol linkers has been demonstrated experimentally. To open future synthetic opportunities, it is compelling to extend the reach of the open-metal site-CH_4_ adsorption strategy by searching for other possible metalated sites that would have optimal CH_4_ adsorptive properties. bpy and cat represent limiting cases of CH_4_ adsorbing coordination compounds: the cat complexes have a “bent” geometry which offers a high number of coordinated CH_4_ molecules, but are very chemically active, while the bpy complexes have lower chemical activity but are not sufficiently attractive and can coordinate no more than two CH_4_ molecules. Other coordination geometries, such as a trigonal (pyramidal) geometry, can potentially be located mid-way, providing high usable capacity and a sufficient attraction and reasonable chemical reactivity. Trigonal coordination could be achieved by using a ligand such the nitrilotriacetic acid (NTA), which is a tri- or tetradentate tripod ligand.^77^ Due to the higher coordination number of the NTA complex with the metal, it is expected to have weaker attractive interactions with CH_4_. Although no MOF containing NTA has yet been prepared, we investigated it here as a model system for evaluating the CH_4_ storage potential of trigonal metal-coordination. We note that a partially coordinated calcium ion is known to exist in nature at the catalytic core of photo-system II.^78^

**Fig. 11:**
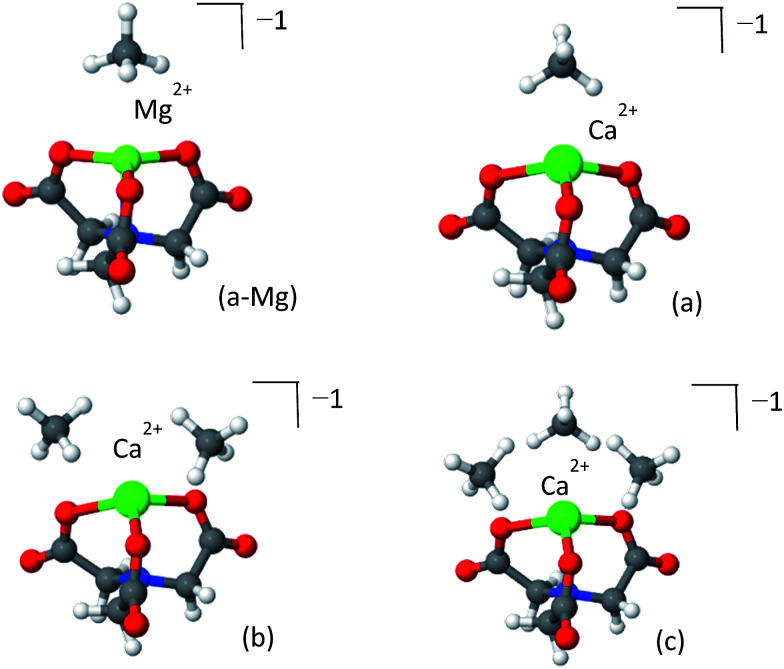
Clusters of NTA-Mg (panel a-Mg) and NTA-Ca (panels a–c) with CH_4_. The first adsorbed CH_4_ NTA-Mg blocks the adsorption site, while three CH_4_ can adsorb to the pyramidal NTA-Ca.

The optimized structures of the NTA-Ca and NTA-Mg are shown in Fig. 11 and the EDA is shown in Table 6. The coordination of the ion with the terminal oxygen atoms forms an exposed sorbent surface above the trigonal planar complex in the case of Mg and an exposed sorbent surface above a trigonal pyramidal complex in the case of Ca. The trigonal pyramidal complex results from the large volume of the Ca^2+^ ion, which prevents the NTA ligand from fully “encapsulating” it, thereby leaving it partially exposed. The EDA indicates that for both NTA-Mg and NTA-Ca, the polarization and charge transfer interactions are the most dominant, as a result of the exposed coordination of the metal.

**Table 6:** Energy Decomposition Analysis (EDA) of the NTA-Ca and NTA-Mg clusters with CH_4_. Values are in kJ mol^−1^

*N*th CH_4_	NTA-ligand			
	ΔFRZ	ΔPOL	ΔCT	Δ*E*_ads_
1 (Mg)	−1.4	−14.8	−5.4	−20.4
1 (Ca)	−2.2	−9.7	−6.3	−17.4
2 (Ca)	−3.4	−8.1	−6.4	−17.0
3 (Ca)	−3.8	−9.1	−6.5	−18.2

**Fig. 12:**
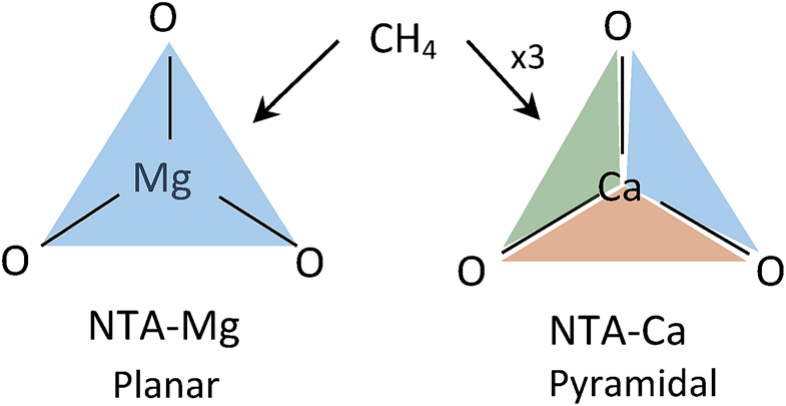
CH_4_ adsorption in planar *vs.* pyramidal exposed surfaces. Each face (colored triangle) can adsorb one CH_4_ molecule.

The pyramidal-exposed geometry of the NTA-Ca complex is a unique feature that can significantly increase its CH_4_ capacity. The pyramidal binding site has three faces, each of which can adsorb a single CH_4_ molecule (Fig. 12). Because the faces of the pyramid are pointed at different directions, the adsorbed CH_4_ molecules are sufficiently separated from each other, by 4.0 Å, such that steric repulsion are minimal. With such small occupancy dependent effects, the bonding characteristics of all adsorbed CH_4_, as seen in the EDA, are very similar, which is clearly promising for sorbent purposes.

Due to the small size of the Mg^2+^ ion, the NTA ligand fully coordinates it to yield a trigonal-planar NTA-Mg complex. The first adsorbed CH_4_ molecule is located on-top of the Mg^2+^ ion, where the induced polarization interactions are the strongest. Being smaller, Mg^2+^ is a better polarizer than Ca^2+^, such that the adsorption energy of a single CH_4_ for NTA-Mg is stronger by −3.0 kJ mol^−1^ and the polarization component is larger by −5.1 kJ mol^−1^ than for NTA-Ca. However, the single methane completely obstructs the binding site for subsequent molecules, such that only one can be effectively adsorbed.

#### Adsorption isotherms and usable site occupancy

**Fig. 13:**
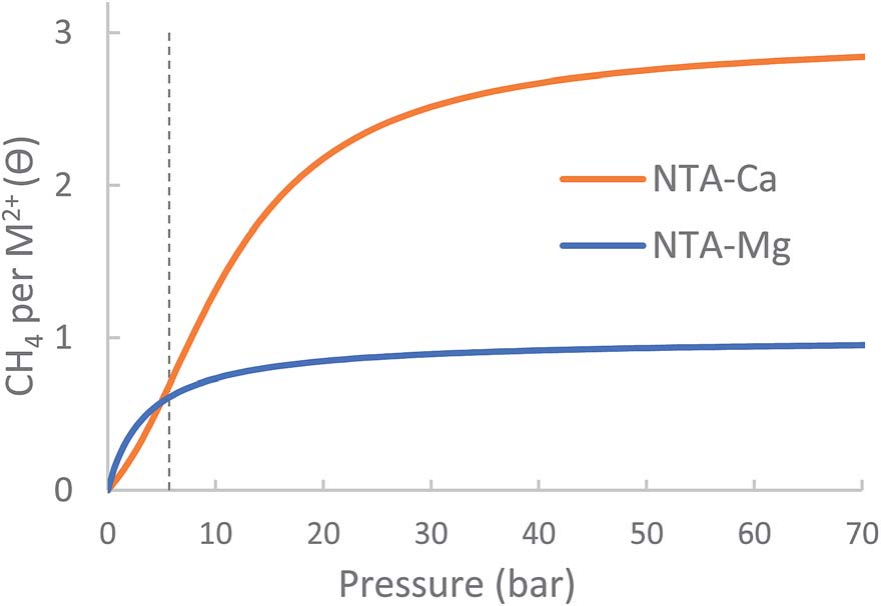
Calculated metal-site adsorption isotherms of the NTA-(Mg, Ca) at 298.15 K.

The metal-site adsorption isotherm of the NTA-Ca and NTA-Mg linkers are shown in Fig. 13 and details of the expected usable site-occupancy are found in Table 7. If NTA-Mg and NTA-Ca could be realized in a MOF, both could significantly contribute to its CH_4_ usable capacity: NTA-Mg has a strong Δ*E*_ads_ of about −20.4 kJ mol^−1^, which results in an expected usable occupancy of 0.29 at 35 bar and 298.15 K, similar to that of MOF-5, due to over-saturation at low pressure. NTA-Ca is found to be even better suited for CH_4_ storage as it adsorbs three CH_4_ molecules with almost similar Δ*E*_ads_ of about −17.5 kJ mol^−1^, which results in a very-high usable site occupancy of 1.90 at 35 bar, more than seven times that of MOF-5.

**Table 7:** Expected metal-site usable occupancy for NTA-(Mg, Ca) 298.15 K. Experimental data for MOF-5 is given for comparison

	NTA-Ca	NTA-Mg	MOF-5
Δ*θ*_uo_ (35 bar)	1.90	0.29	1.6
Δ*θ*_uo_ (65 bar)	2.12	0.33	2.4

Since no NTA-linker based MOF is known to exist, usable capacity estimates for this open metal site embedded in a framework cannot not be provided. However, the NTA-Ca system clearly demonstrates the tremendous potential of using partially coordinated calcium ion for the purpose of CH_4_ storage.

### The effect of solvent on methane adsorption

Although cat metalated linkers have good CH_4_ adsorption abilities, their realization in experiment could face difficulties due to the high reactivity of the almost completely exposed metal ion centre. As already explained above, the exposed ions can attract guest molecules that may be deposited in the MOF during its preparation and processing, such as solvent molecules. These species are attracted to the metal ion several times more strongly than CH_4_, thereby effectively poisoning it toward CH_4_ adsorption. Furthermore, the removal of a typical solvent molecule from cat-(Mg, Ca) would require energies of about −100 kJ mol^−1^ (the binding energy of methanol and acetonitrile), which is much higher than the thermal energy present at room temperature, or even at 150–250 °C, and approaches the thermal stability limit of most MOFs. Hence the presence of solvent molecules is difficult to avoid, and would be typically viewed as a significant potential deterrent to pursuing the synthetic realization of such sites.

However, we would like to point out a different, and potentially much more optimistic perspective. The presence of solvent molecules at the open-metal site could actually be advantageous. The exposed metal ions will be attracted to the negatively charged component of the MOF, thus decreasing the overall framework stability, but the presence of residual solvent molecules could potentially stabilize the MOF by making the metal centre more chemically viable. Considering these prospects, it is intriguing to explore the effect of the presence of solvent on the adsorption of CH_4_. Here, we study the CH_4_ adsorption of cat-(Mg, Ca) in the presence of a single methanol (MeOH) and acetonitrile (MeCN) solvent molecules, which are often used in the final washing step of the MOF before its activation. Note that, due to the large number of possible solvents and number of solvent molecules, a more thorough study of solvent effects is beyond the scope of this work.

**Fig. 14:**
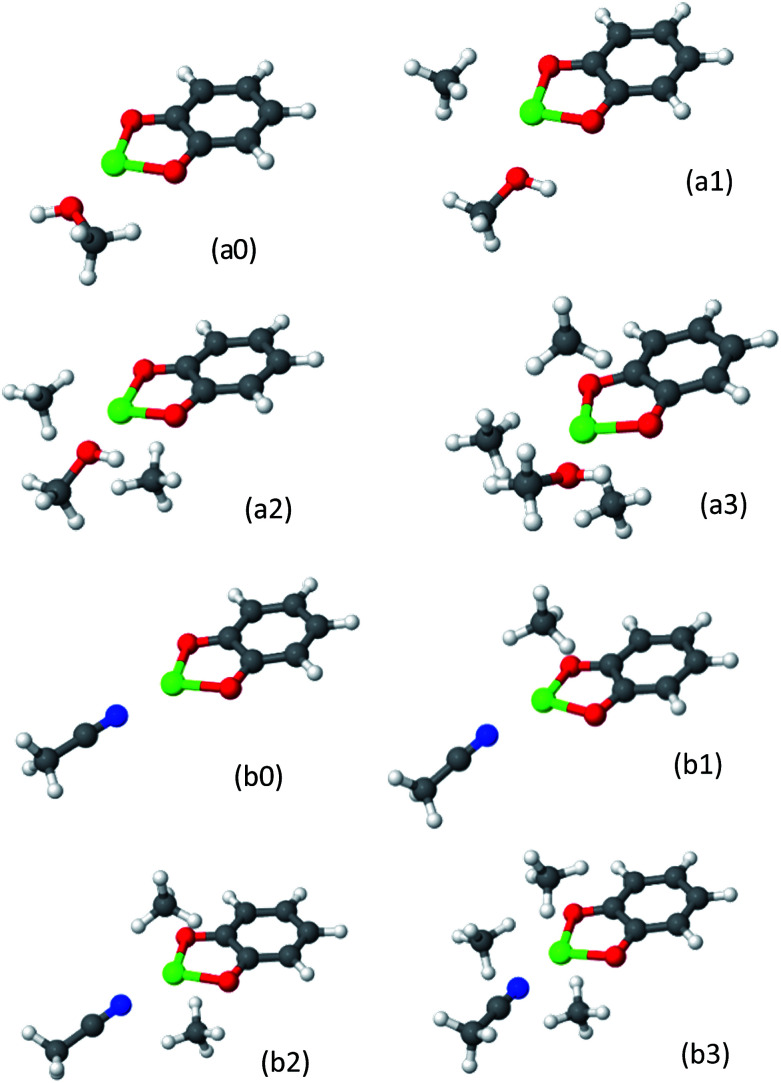
CH_4_ adsorbed on metalated cat-Mg linkers in the presence of solvent molecule. Panels (a0–a3) and (b0–b3) show MeOH and MeCN solvents-complexes, consecutively. As one solvent molecule is coordinated to the metal, no more than three CH_4_ molecules can have significant contribution to the CH_4_ usable site occupancy.

The resulting structures for cat-Mg are shown in Fig. 14, and the EDA of the clusters and of the solvent–metal interaction, compared to the solvent-free complex, are shown in Table 8. The structures of cat-Ca are given in the ESI.†

Effect of solvent on CH_4_ adsorption: comparison of the polarization component of the EDA and total energies of CH_4_ interaction with solvent@cat-(Mg, Ca). — no solvent, MeCN – acetonitrile, MeOH – methanol. The frozen and CT interactions are not given, as they are not considerably affected by the solvent's presence. All values are in kJ mol^−1^

**Table:** 

*N*(CH_4_)	Cat-Mg					
	Δ*E*_POL_			Δ*E*_ads_		
	—	MeOH	MeCN	—	MeOH	MeCN
1	−49.9	−36.7	−21.1	−40.7	−20.7	−17.3
2	−33.6	−13.2	−13.0	−27.0	−19.9	−15.5
3	−17.9	−4.2	−3.6	−14.6	−16.5	−12.5
Total	−101.4	−54.2	−37.7	−82.3	−57.1	−45.3

**Table:** 

*N*(CH_4_)	Cat-Ca					
	Δ*E*_POL_			Δ*E*_ads_		
	—	MeOH	MeCN	—	MeOH	MeCN
1	−19.9	−18.2	−16.7	−26.7	−23.2	−22.6
2	−17.6	−15.5	−14.4	−24.7	−22.5	−17.6
3	−16.0	−13.8	−13.9	−21.7	−20.3	−21.0
Total	−53.5	−47.6	−45.0	−73.2	−65.9	−61.2

The maximum number of CH_4_ molecules that are substantially attracted to the solvent@cat-(Mg, Ca) complex is three, compared to four when no solvent is present. This implies that, as expected, the solvent molecule occupies a single site in the metal ion coordination sphere.

The comparison of the EDA of cat-Mg and cat-Ca sheds light on the effect of the solvent. The solvent shields the ion and prevents it from inflicting strong polarizing forces on the adsorbed CH_4_ molecule. The shielding effect is stronger for cat-Mg than for cat-Ca, since Mg^2+^ is smaller than Ca^2+^, it tends to polarize its environment and is more easily shielded. As can be seen in Table 8, for cat-Mg the adsorption energy (Δ*E*_ads_) for three adsorbed CH_4_ molecules in the presence of MeOH, is reduced by −25 kJ mol^−1^, but only by −7 kJ mol^−1^ for cat-Ca. The EDA also shows that this large reduction for cat-Mg is mostly due to a −47 kJ mol^−1^ decrease in the polarization component, as a result of solvent shielding.

#### Solvent effect on CH_4_ usable site occupancy

**Fig. 15:**
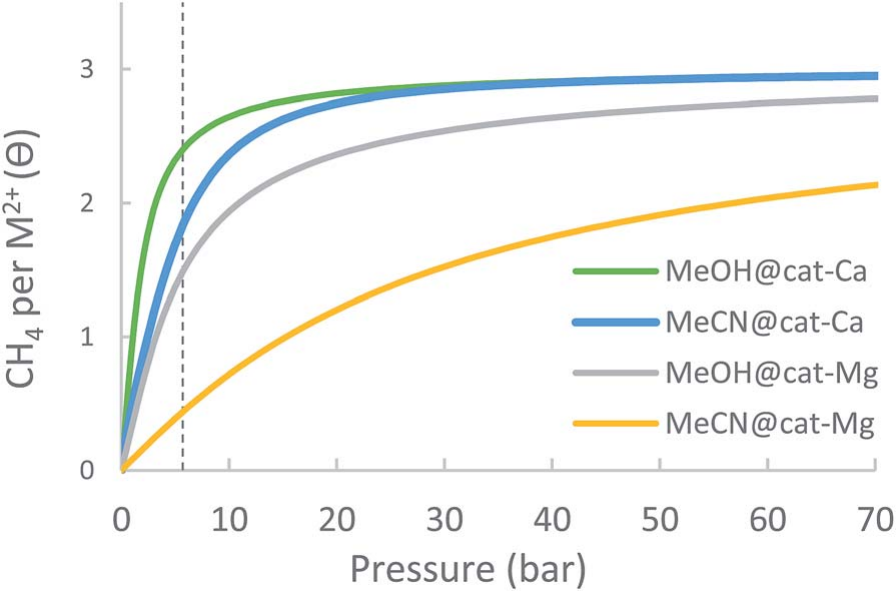
Calculated adsorption isotherms of cat-(Mg, Ca) metal-site with coordinated solvent molecule (MeOH/MeCN) at 298.15 K.

The adsorption isotherm of cat-(Mg, Ca) metalated linkers, in the presence of MeOH and MeCN are shown in Fig. 15 and details of the expected usable capacities are found in Table 9. Although the presence of solvent generally reduces the adsorption energy, the usable site occupancy of the MOFs is not necessarily reduced and can even increase. Indeed, for cat-Ca, it is found that the presence of solvent molecules can significantly increase the usable site occupancy: the solvent attenuates the electrostatic fields induced by the Ca^2+^ ion, thereby reducing its interaction with CH_4_ into the optimal range for storage at ambient conditions. Thus, usable site occupancy of the metal site cat-Ca in the presence of MeCN is expected to increase by 0.69 at maximum pressure of 35 bar, with respect to the solvent-free case. For cat-Mg in the presence of MeCN and MeOH, it is found that the usable site occupancy can slightly increase (MeCN) or decrease (MeOH) with respect to the non-solvated case, indicating that this metal-site is resilient to solvent poisoning. These results demonstrate an important finding, that solvent can be used to attenuate overly strong sorbent–host interactions and thus improve usable capacity in cases where the occupancy of the open-metal sites is high at low pressures. Of course these conclusions depend upon achieving at least partial desolvation of the open-metal site – a fully blocked site is fully poisoned, but partial desolvation is typically much more tractable than complete desolvation.

Expected usable metal-site occupancy for cat-(Mg, Ca) in the presence of MeOH and MeCN solvent molecules 298.15 K. Data for MOF-5 is given for comparison

**Table:** 

	Cat-Mg	MeOH@cat-Mg	MeCN@cat-Mg	MOF-5
Δ*θ*_uo_ (35 bar)	1.13	1.10	1.20	1.6
Δ*θ*_uo_ (65 bar)	1.47	1.26	1.64	2.6

**Table:** 

	Cat-Ca	MeOH@cat-Ca	MeCN@cat-Ca	MOF-5
Δ*θ*_uo_ (35 bar)	0.34	0.49	1.03	1.6
Δ*θ*_uo_ (65 bar)	0.37	0.53	1.09	2.6

## Discussion and conclusion

### Expected usable capacity of linker-metalated MOFs

From estimating the properties of the individual metal-site, we now proceed to estimating the potential contribution to the usable capacity of actual MOFs. The reader is provided with rough estimates of the usable capacity of MOFs modified to accommodate open-metal sites of the type studied here. We use existing experimental data for pre-metalated MOFs and estimate its post-modification usable capacity. Particularly, MOF-5 (Zn_4_O(bdc)_3_) is used to demonstrate the increase in usable capacity by catechol linker metalation and UiO-67-bpy (Zr_6_O_4_(OH)_4_(bpydc)_6_) to demonstrate bpy linker metalation.

We assume that the linkers of the parent MOF had been metalated or completely replaced by new metalated-linkers. It is also assumed that the volume and surface area of the parent MOF are identical to the modified MOF and that the contribution of new open-metal sites to the overall CH_4_ storage, is additive to the capacity of the parent MOF. This idealized approach allows us to provide an upper bound on the usable capacity. Of course the actual numbers must be smaller, due to reduction in the MOF surface area upon metalation, possible steric overlap between CH_4_ molecules adsorbed on different sites and steric repulsion from the MOF itself, especially at high site-occupancy. There can also be significant materials limitations, such as the fact that we employ the crystalline density, whereas the material at present is only available as a powder at densities that are several times lower, as demonstrated for MOF-5.^79^ However, the important question is to identify the upper limit to potential usable capacity, and thus the motivation for future experimental efforts.

The maximal addition of usable CH_4_ capacity (Δ*n*_oms_), in v[STP]/v units, as a result of the introduction of the open-metal sites to the structure is evaluated as follows:5
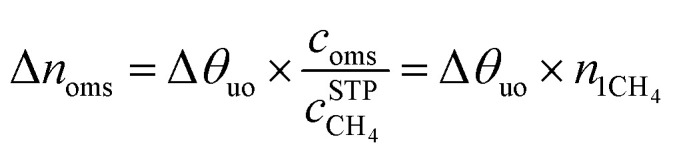
where Δ*θ*_uo_ is the usable site occupancy, *c*_oms_ is the molar concentration of the open-metal sites and *c*_CH_4__^STP^ is the molar concentration of CH_4_ at standard conditions (25 °C, 1 atm). The quantity *n*_1CH_4__ is the amount of methane adsorbed in the MOF, in v/v units, if a single CH_4_ molecule occupies the open-metal site. Further information regarding this estimate is found in the ESI.†

The main results are shown in Table 10. As seen in the last row of Table 10, several metalated MOFs can potentially cross the current limitation of 200 v/v at 65 bar. Metalation by cat-Mg is the most beneficial with a contribution of about 60 v/v at 35 bar. At higher pressure of 65 bar this contribution increases by about 18 v/v. Due to the small usable site occupancy, metalation of UiO-67-bpy by CaCl_2_ results in a small increase of 26 v/v at 35 bar. At higher pressure this contribution is increased only to about 28 v/v such that the total expected usable capacity of UiO-67-bpy-CaCl_2_ at 65 bar is about 191 v/v.

**Table 10:** Expected usable capacities for MOFs modified with open-metal sites. bdc2− = 1,4-benzenedicarboxylate, bpydc2− = 2,2′-bipyridine-5,5′-dicarboxylate. In all cases minimal pressure (*P*_min_) is 5.8 bar. The expected capacity is the sum of the parent capacity and the metalated linker capacity

	MOF-5	MOF-5	MOF-5	UiO-67-bpy
Parent linker	bdc	bdc	bdc	bpydc
Modified linker	Cat-Mg	Cat-Ca	MeCN@cat-Ca	bpy-CaCl_2_
Parent capacity [v/v] 35 bar	114	114	114	115
Metalated linker capacity [v/v] 35 bar	60	18	54	26
Expected capacity [v/v] 35 bar	174	132	168	141
Parent capacity [v/v] 65 bar	179	179	179	163
Metalated linker capacity [v/v] 65 bar	78	19	58	28
Expected capacity [v/v] 65 bar	257	198	237	191

The usable capacity of MOF-5-cat-Ca can be improved if a residual solvent molecule (MeCN) is present, which increases its usable capacity by 36 v/v, from 132 v/v to 168 v/v at 35 bar. At maximum pressure of 65 bar the usable capacity of MOF-5-cat-Ca@MeCN can possibly reach 237 v/v.

The main implications of these numbers is that material designs of these types have considerable potential, even allowing for the inevitable limitations of real materials, including lack of monolithic single crystals, residual solvent and other trace contaminants in real natural gas. Finally, while designing an adsorbent that can operate at ambient temperature is preferred, we note that these materials with strong CH_4_ binding sites would benefit significantly from using waste heat on-board a vehicle to maximize the usable capacity by increasing the desorption temperature.

## Conclusion

Metal–organic frameworks (MOFs) offer tremendous surface area for gas storage, but the simultaneous demands of high surface area, adequate thermal and mechanical stability, and the ideal binding energies for multiple methane molecules at a single site are nearly impossible to satisfy. Rather, it may be better to divide and conquer, and address the binding site requirements by post-synthetic modifications, such as the introduction of open-metal sites, as decorations to relatively rigid organic linkers. This work has been concerned with the possibilities and limitations of such sites for the purposes of methane storage. Our main conclusions are as follows:

(1) Adequate interaction energy is a necessary condition for an effective CH_4_ storage. The results obtained here imply, that in order to have a sufficient CH_4_ adsorption ability, the metal ion should not only be (partially) exposed, but should also have sufficient ionic character, *i.e.*, the charge separation of the metal ion and its coordination environment should be large. However, ionic character in molecules is closely related to deviation from covalency, which tends to spread the electronic densities in between the atoms, preventing accumulation of charge on specific atoms. Preparation of such species poses considerable synthetic challenges: for instance, for the bpy linker, only the bpy-CaCl_2_ complex has significant interaction with CH_4_—which is made possible by its weak coordination by the linker, which could prevent the Ca^2+^ ion from forming a stable complex. In cat-Mg and cat-Ca, which both have promising CH_4_ adsorption properties, the metal ion is almost completely exposed, being coordinated by only two oxygen atoms. Cat-Mg and cat-Ca are therefore expected to be highly reactive and can potentially affect the MOFs structural stability.

(2) While adequate interaction energy is a necessary condition for CH_4_ adsorption, the overall uptake of the open-metal site is also related to the site geometry, where pyramidal, as in NTA-Ca, or “bent”, as in cat-(Mg, Ca), can accommodate more CH_4_ molecules that are adsorbed with approximately the same energy. The formation of geometrically exposed sites, where the coordination sphere of the metal-ions is exposed along more than one axis, can result in a substantial increase in the capacity of the site.

(3) The usable capacity of the catechol based cat-Mg open-metal sites benefits mostly from the large number of coordinated methane molecules: although the first adsorbed CH_4_ is bound too strongly, the subsequent ones are more weakly bound and contribute to effective adsorption. Even in the presence of solvent which greatly reduces the adsorption energy, the overall usable capacity remains similar, or increases. Too strongly bound CH_4_ molecules in the non-solvated case become storage-effective in the presence of solvent, which compensates for the reduced effectiveness of the more weakly attracted CH_4_.

(4) Another important point is the ratio between total adsorbed CH_4_ and the storage-usable adsorbed CH_4_. Since effectively not much more than a single CH_4_ contributes to the usable capacity of the cat-(Ca, Mg) site, their usable capacity is low with respect to their total capacity, such that a significant portion of the volume of the MOF is expected to be “wasted” on non-usable capacity. For instance, cat-Mg has a ratio of only 1 : 4 usable to total adsorbed CH_4_. The bpy-CaCl_2_ and NTA-Ca open-metal sites are more effective in that sense, where relatively more methane molecules per open-metal site contribute to the usable capacity of the MOF and the ratio between usable and total adsorbed CH_4_ is higher. NTA-Ca is especially effective with a ratio of 9 : 10 usable to total adsorbed CH_4_.

(5) Another interesting finding is the unique properties of the Ca^2+^ ion: for some systems, calcium is very resilient to its coordination environment and is able to maintain its CH_4_ adsorbing properties in the presence of solvent or when coordinated by the tridentate NTA ligand. This seems to be a result of its large ionic radius, such that the coordinating environment is not able to shield its electrostatic charge, which is dispersed over a large area. When the calcium ion remains partially exposed it can adsorb one or more CH_4_ with more-than sufficient energy, depending on the steric availability in its coordination sphere.

(6) While the exact effect of solvent molecule(s) is difficult to determine and specific recommendations cannot be given, this study demonstrates that the presence of residual solvent is not necessarily detrimental for CH_4_ adsorption applications. On the contrary, solvent molecules can be used to modify the interaction between the open-metal site and the adsorbed CH_4_ molecule, especially for attenuating excessively strong binding into the desired range. The large variety of solvent molecules implies that other effects could be expected for the complex-solvent–CH_4_ system. These are left for future investigations, perhaps in response to future specific experimental developments in the field.

## Supplementary Material

SC-007-C6SC00529B-s001
