# Supplementary material for: A computational study of CH4 storage in porous framework materials with metalated linkers: connecting the atomistic character of CH4 binding sites to usable capacity
Source: Chem Sci. 2016 Mar 29;7(7):4503–18. doi: 10.1039/c6sc00529b (PMC6016331; doi:10.1039/c6sc00529b)
Supplement: SC-007-C6SC00529B-s001 [file SC-007-C6SC00529B-s001.pdf]

Electronic Supplementary Material (ESI) for Chemical Science.
This journal is © The Royal Society of Chemistry 2016

Supporting Information

A Computational Study of CH_4 Storage in Porous Framework Materials with Metalated Linkers: Connecting the Atomistic Character of CH_4 Binding Sites to Usable Capacity

Ehud Tsivion,[†, §] Jarad A. Mason,[§] Miguel I. Gonzalez,[§] Jeffrey R. Long,[†, §, #] Martin Head-Gordon[‡, §, *]

† Materials Sciences Division and ‡ Chemical Sciences Division, Lawrence Berkeley National Laboratory, Berkeley, California 94720, United States.

§ Department of Chemistry and # Department of Chemical and Biomolecular Engineering, University of California, Berkeley, California 94720, United States

Table of Content

Evaluation of CH_4 capacity of metalated MOF-5 and UiO-67-bpy 2
Representation of the parent MOF usable capacity 3
Optimized structures of solvent@catechol-Ca 4
The effect of adsorption entropy (ΔS_{ads}) on expected capacities 5
Full Energy Decomposing Analysis (EDA) tables 7
Synthesis of $Zr_6O_4(OH)_4(bpydc)_6$ ($bpydc^{2-}$ = 2,2′-bipyridine-5,5′-dicarboxylate) 8
High-Pressure CH_4 Adsorption 8
Comparison to experiment: CH_4 adsorption of in MOF-5 10
References 13

Evaluation of CH_4 capacity of metalated MOF-5 and UiO-67-bpy

The following section explains the reasoning and assumptions behind the estimation of the capacities of child-MOFs derived from parent-MOFs by the addition of open-metal sites (OMSs). The parent MOFs that are studied are MOF-5,(1) to be modified with catechol (cat) linkers metalated with Ca and Mg ions, and UiO-67-bpy(2) where the bipyridine (bpy) linkers are metalated with MX_2 type metals (M^{2+}) and counter-ions (X^-). The following approximations are assumed:

1. There is no material density loss due to imperfect packing of the material.
2. Pore window size allow for free diffusion of the CH_4 molecules in the material.
3. No overlap between CH_4 adsorbed on the OMS adsorbed and CH_4 adsorbed in other places.
4. 100% percent linker metalation.

The maximal addition of usable CH_4 capacity (Δn_{oms}), in v[STP]/v units, as a result of the introduction of the open-metal sites to the structure of the parent MOF is evaluated as follows:

$$\Delta n_{oms} = \Delta\theta_{uo} \times \frac{c_{oms}}{c_{CH_4}^{STP}} = \Delta\theta_{uc} \times n_{1CH4}$$

Where $\Delta\theta_{uo}$ is the usable site-occupancy, c_{oms} is the molar concentration of the open-metal sites in the parent MOF, $c_{CH_4}^{STP}$ is the molar concentration of CH_4 at standard conditions (25 °C, 1 atm). The quantity n_{1CH4} corresponds to the amount of methane adsorbed in the MOF if a single CH_4 molecule occupies the open-metal site, in v[STP]/v units. Calculation of $\Delta\theta_{uo}$, the usable site-occupancy, is described in detail in the paper.

Assuming 100% metalation of the linkers, the open-metal sites concentration, c_{oms}, is equal to the concentration of the linkers c_{linker}, i.e. $c_{oms} = c_{linker}$. The linker/OMS concentration is evaluated as follows:

$$c_{linker} = \frac{N_{linker}}{V_{cell} \cdot N_A}$$

where V_{cell} is the volume of the unit cell in $[L]$ units, N_A is Avogadro's number in $[mol^{-1}]$ units and N_{linker} is the number of linkers in a unit-cell.

The concentration of linker in the parent MOF-5 and UiO-67-bpy, as well as other relevant values, is listed in the following table:

	MOF-5	UiO-67-bpy
V_{cell} [Å^3]	16913.24	18651.79
N_{linker}	24	24
c_{linker}[mol/L]	2.36	2.14
n_{1CH4} v[STP]/v	52.79	47.87

The maximal expected usable capacity of a child MOFs (n_{child}), as a result of the introduction of open-metal site into it parent MOF, is estimated using:

$$n_{child} = n_{parent} + n_{oms}$$

The experimental data, including the usable capacity of the parent MOFs n_{parent}, for MOF-5 and UiO-67-bpy is reported by Mason et al.(3) and here in section "High-Pressure CH_4 Adsorption" respectively. For the parent MOFs the usable capacity is evaluated using the CH_4 total adsorption isotherms at 25 °C, fitted to a single-site Langmuir equation:

$$n_{ads}(p) = n_{sat} \times \frac{K \cdot p}{1 + K \cdot p}$$

where $n_{ads}(p)$ is the amount of CH_4 adsorbed in the parent MOF at a given pressure p, in v[STP]/v units. n_{sat} is the capacity of the parent MOF at full saturation in v [STP]/v units, and K is the Langmuir parameter in bar^{-1}. The fitted parameters obtained are:

	K	n_{sat}	n_{parent} v[STP]/v 5.8-35 bar	n_{parent} v[STP]/v 5.8-65 bar
MOF-5	0.0146	440	114	180
UiO-67-bpy	0.0287	321	115	163

Representation of the parent MOF usable capacity

The non-metalated parent MOFs have substantial CH_4 capacity by their own, dictated by their structure and composition. Methane adsorption in the parent MOFs also occurs on well-defined centers, most likely on the metal-clusters (or just "clusters"), which are more reactive with respect to the (non-metalated) linkers, as evident from previous experimental and computational studies.

To increase the readability of the paper, we compare the expected usable site occupancy of the open-metal sites to that of the clusters in the parent MOFs. This is done by rewriting the Langmuir equation, to obtain $\theta_{cluster}$, cluster occupancy values that are comparable to θ_{oms}, the occupancy of the open-metal sites. However, the metal clusters contain several metal ions, therefore for a balanced comparison $\theta_{cluster}$ is evaluated per metal-ion in the cluster, such that metal in the clusters are compared vs. the metals on the linkers.

In the new representation, each metal in the cluster can adsorb θ_{max} number of methane molecules, each of which contributes $n_{cluster}$ to the MOF capacity. The Langmuir equation takes the following form:

$$n_{ads}(p) = n_{sat} \times \frac{K \cdot p}{1 + K \cdot p} = n_{1CH4\_cluster} \times \theta_{max} \times \frac{K \cdot p}{1 + K \cdot p} = n_{1CH4\_cluster} \times \theta_{cluster}(p)$$

The quantity $n_{1CH4\_cluster}$ corresponds to the amount of methane adsorbed in the MOF if a single CH_4 molecule occupies the one metal ion in the cluster (cluster-metal). This number is different from n_{1CH4} of the open-metal site, since the number/concentration of cluster-metals is different from the number of open-metal sites. Since the value of the Langmuir expression $\frac{K \cdot p}{1+K \cdot p}$ swings between zero and one, the value of $\theta_{cluster}$ swings between zero (cluster-metal is completely empty) and θ_{max} (cluster-metal is occupied by θ_{max} number of methane molecules). Since fitting to the single-site Langmuir model is an approximation, the value of θ_{max} is not necessarily an integer and is straightforwardly given by:

$$\theta_{max} = {}^{n_{sat}}/_{n_{1CH4\_cluster}}$$

The values obtained for the parent MOFs are:

	MOF-5	UiO-67-bpy
$N_{cluster-metals}$	32	24
$n_{1CH4\_cluster}$ v[STP]/v	70.38	47.87
θ_{max}	6.25	6.7

Optimized structures of solvent@catechol-Ca

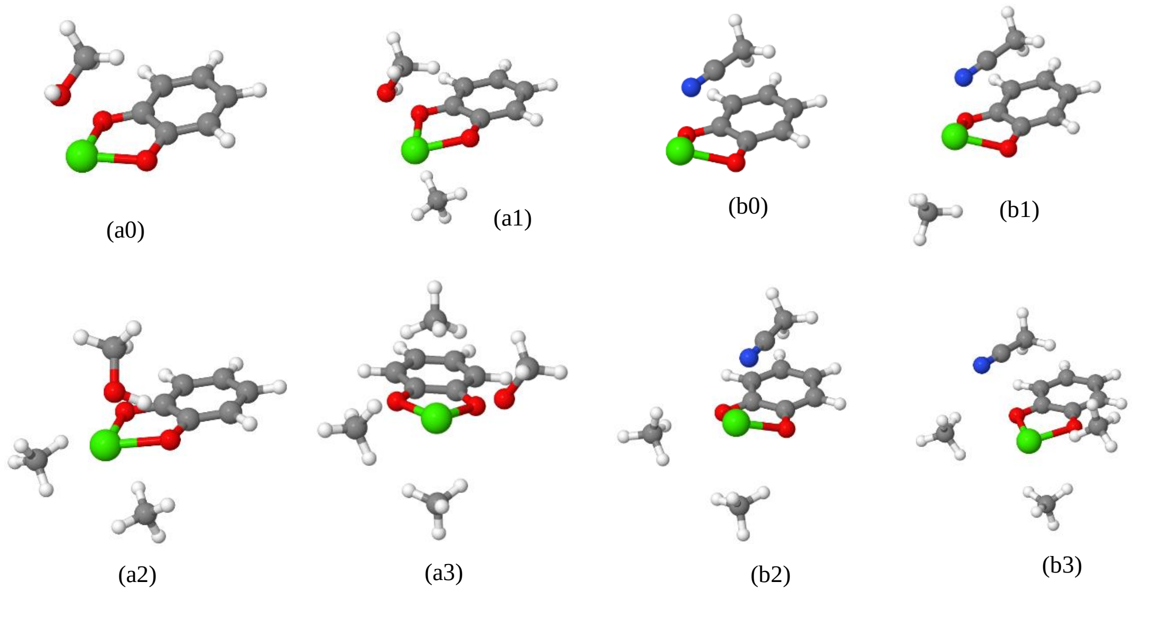

Figure S1. CH_4 adsorbed on metalated cat-Ca linkers in the presence of solvent molecule. Panels (a0-a3) and (b0-b3) show MeOH and MeCN solvents-complexes, consecutively. As one solvent molecule is coordinated to the metal, no more than three CH_4 molecules can have significant contribution to the CH_4 capacity of the MOF at ambient conditions.

The effect of adsorption entropy (ΔS_{ads}) on expected capacities

Since accurate evaluation of the entropy of adsorption (ΔS_{ads}) is currently not computationally feasible (as explained in the paper), the adsorption entropy was assumed to be approximately constant for all adsorbed CH_4 molecules. The value of ΔS_{ads} = −9.5 kJ mol^{-1} K^{-1} which represent an intermediate of values measured for materials for adsorptive storage applications.(3,4) Here, we study the implications of this assumption by looking at two other options for evaluating ΔS_{ads}. In the first case the entropy is taken to be ΔS_{max} = −10.0 kJ mol^{-1} K^{-1} which is a rather large value that was measured for CH_4 adsorption in Ni_2(dobdc). For the second case we assume that there exists a relation between ΔH and ΔS such that stronger adsorption enthalpies result in larger changes in entropies, ΔS is estimated using a linear calibration curve based on previously obtained data for the first CH_4 adsorption sites for several MOFs (

Table S1).

MOF name	ΔH	ΔS
AX-21	−10.7	−9.20
MOF-5	−12.3	−9.20
HKUST-11	−17.1	−9.70
Mg_2(dobdc)	−18.6	−9.60
Co_2(dobdc)	−19.7	−9.70
Ni_2(dobdc)	−21	−10.00

Table S1. Adsorption enthalpies of entropies measured for different MOFs.

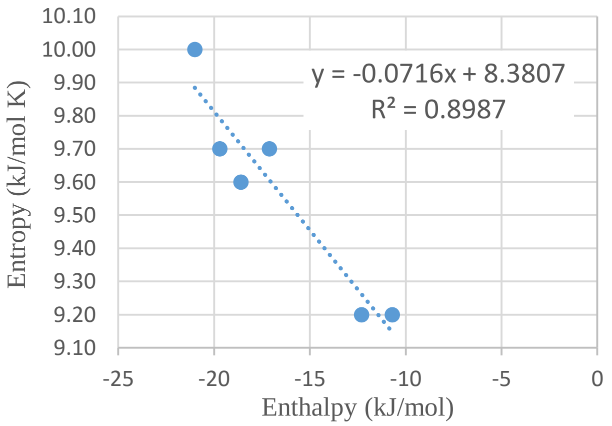

Figure S2. Calibration curve for fitting ΔS to ΔH.

Overall, for most of the cases studied in the article which result in significant CH_4 usable capacity, increasing the values of ΔS_{ads} doesn't significantly affect the nature of the results, and in some cases results in higher capacities due to relaxation of over-bounded CH_4 at low pressures. Exact numbers are given below at Table S2.

Table S2. Comparison of various ΔS_{ads} on the differential occupancy.

	Δθ (35 bar)
Cat-Mg	1.13
Cat-Mg-ΔSmax	0.89
Cat-Mg-ΔSfit	1.15

MeOH@cat-Mg	1.10
MeOH@cat-Mg-ΔSmax	1.32
MeOH@cat-Mg-ΔSfit	1.29

MeCN@cat-Mg	1.20
MeCN@cat-Mg-ΔSmax	0.97
MeCN@cat-Mg-ΔSfit	1.27

Cat-Ca	0.34
Cat-Ca-ΔSmax	0.59
Cat-Ca-ΔSfit	0.58

MeOH@cat-Ca	0.49
MeOH@cat-Ca-ΔSmax	0.75
MeOH@cat-Ca-ΔSfit	0.71

MeCN@cat-Ca	1.03
MeCN@cat-Ca-ΔSmax	1.44
MeCN@cat-Ca-ΔSfit	1.29

NTA-Ca	1.90
NTA-Ca-ΔSmax	1.86
NTA-Ca-ΔSfit	1.92

NTA-Mg	0.29
NTA-Mg-ΔSmax	0.36
NTA-Mg-ΔSfit	0.34

bpy-CuCl2	0.32
bpy-CuCl2-ΔSmax	0.21
bpy-CuCl2-ΔSfit	0.40

bpy-ZnCl2	0.15
bpy-ZnCl2-ΔSmax	0.09
bpy-ZnCl2-ΔSfit	0.22

bpy-CaCl2	0.54
bpy-CaCl2-ΔSmax	0.82
bpy-CaCl2-ΔSfit	0.74

Full Energy Decomposing Analysis (EDA) tables

Cat-Mg

	ΔFRZ	ΔPOL	ΔCT	ΔGD	ΔTotal
1	10.0	-49.9	-6.3	5.5	-40.7
2	13.7	-33.6	-8.9	1.8	-27.0
3	11.2	-17.9	-8.2	0.3	-14.6
4	-12.2	-1.0	-5.7	1.8	-17.1

Cat-Mg-methanol

	ΔFRZ	ΔPOL	ΔCT	ΔGD	ΔTotal
1	8.9	-36.7	-6.5	13.6	-20.7
2	0.5	-13.2	-8.2	1.0	-19.9
3	-8.0	-4.2	-4.2	-0.1	-16.5

Cat-Mg-acetonytril

	ΔFRZ	ΔPOL	ΔCT	ΔGD	ΔTotal
1	7.3	-21.1	-6.9	3.4	-17.3
2	-4.6	-13.0	-4.3	6.4	-15.5
3	-3.4	-3.6	-4.3	-1.2	-12.5

Cat-Ca

	ΔFRZ	ΔPOL	ΔCT	ΔGD	ΔTotal
1	0.8	-19.9	-10.3	2.6	-26.7
2	0.8	-17.6	-8.5	0.6	-24.7
3	0.1	-16.0	-7.6	1.7	-21.7
4	0.1	-16.4	-7.8	1.9	-22.2

Cat-Ca-Methanol

	ΔFRZ	ΔPOL	ΔCT	ΔGD	ΔTotal
1	-0.2	-18.2	-9.0	4.2	-23.2
2	-0.9	-15.5	-7.5	1.5	-22.5
3	0.1	-13.8	-9.2	2.6	-20.3

Cat-Ca-Acetonytril

	ΔFRZ	ΔPOL	ΔCT	ΔGD	ΔTotal
1	0.6	-16.7	-8.0	1.4	-22.6
2	-1.3	-14.4	-7.5	5.7	-17.6
3	-1.5	-13.9	-7.3	1.6	-21.0

NTA-Ca

	ΔFRZ	ΔPOL	ΔCT	ΔGD	ΔTotal
1	-2.2	-9.7	-6.3	0.8	-17.4
2	-3.4	-8.1	-6.4	0.8	-17.0
3	-3.8	-9.1	-6.5	1.1	-18.2

NTA-Mg

	ΔFRZ	ΔPOL	ΔCT	ΔGD	ΔTotal
1	-1.4	-14.8	-5.4	1.2	-20.4

bpy-CuCl2

	ΔFRZ	ΔPOL	ΔCT	ΔGD	ΔTotal
1	-7.1	-2.8	-3.2	0.2	-12.9
2	-4.3	-4.1	-3.7	1.9	-10.2

bpy-ZnCl2

	ΔFRZ	ΔPOL	ΔCT	ΔGD	ΔTotal
1	-3.9	-2.6	-3.8	0.1	-10.1
2	-3.9	-2.5	-3.9	0.3	-10.1

bpy-CaCl2

	ΔFRZ	ΔPOL	ΔCT	ΔGD	ΔTotal
1	-1.2	-11.8	-9.5	-21.1	-19.8
2	-3.9	-11.4	-9.5	-23.7	-21.3

Synthesis of $Zr_6O_4(OH)_4(bpydc)_6$ ($bpydc^{2-}$ = 2,2′-bipyridine-5,5′-dicarboxylate)

The compound $Zr_6O_4(OH)_4(bpydc)_6$, which is referred to as UiO-67-bpy in this work, was synthesized as previously reported.(5)

High-Pressure CH_4 Adsorption

The high-pressure adsorption isotherm for UiO-67-bpy was measured on a HPVA-II-100 from Particulate Systems, a Micromeritics company. Here, activated sample was loaded into a tared 2 mL stainless steel sample holder inside a glovebox under a N_2 atmosphere. Prior to connecting the sample holder to the VCR fittings of the complete high-pressure assembly inside the glove box, the sample holder was weighed to determine the sample mass. The sample holder was then transferred to the HPVA-II-100, connected to the instrument's analysis port via an OCR fitting, and evacuated at room temperature for at least 1 h. The sample holder was placed inside an aluminum recirculating dewar connected to a Julabo FP89-HL isothermal bath filled with Julabo Thermal C2 fluid. The temperature stability of the isothermal bath is ± 0.02 °C. Methods for accurately measuring the sample freespace, which involve the expansion of He from a calibrated volume at 0.7 bar and 25 °C to the evacuated sample holder, were described in detail previously.(3) Nonideality corrections were performed using the CH_4 compressibility factors tabulated in the NIST REFPROP database for each measured temperature and pressure.(6)

The experimentally measured excess amounts adsorbed were converted to total amounts adsorbed using the equation below, where n_{ex} is the excess amount adsorbed in mmol/g, n_{tot} is the total amount adsorbed in mmol/g, V_p is the pore volume in cm^3/g, and ρ_{bulk} is the bulk density of pure CH_4.

$$n_{tot} = n_{ex} + V_p \cdot \rho_{bulk}(P,T)$$

The NIST Refprop database was used to determine ρ_{bulk} at each temperature and pressure.(6) The total pore volume of UiO-67-bpy was determined from a previously 77 K N_2 adsorption isotherm to be 0.99 cm^3/g from the an uptake of mmol/g at P/P_0 of 0.9. The crystallographic density of UiO-67-bpy is 0.747 g/cm^3.

Note that the unit v/v is equivalent to cm^3_{STP} cm^{-3}, where cm^3_{STP} is defined as the volume occupied by an ideal gas at standard temperature and pressure (STP). Here, STP is defined as 273.15 K and 1 atm, resulting in a volume of 22.414 mL for 1 mmol of ideal gas at STP.

Table S3. High-pressure CH_4 adsorption data for UiO-67-bpy at 25 °C.

Pressure (bar)	Excess CH_4 adsorbed (mmol/g)	Total CH_4 adsorbed (mmol/g)	Total CH_4 adsorbed (v/v)
0.9	0.4	0.5	7.6
2.1	1.0	1.1	17.7
4.1	1.8	2.0	33.4
5.8	2.5	2.7	45.6
7.8	3.2	3.5	58.7
10.1	3.9	4.3	72.4
12.6	4.6	5.1	85.6
15.2	5.2	5.9	98.0
20.1	6.2	7.0	117
25.1	7.0	8.1	135
30.1	7.6	8.9	149
35.1	8.2	9.6	161
40.0	8.5	10.2	172
45.2	8.9	10.8	181
50.0	9.2	11.3	190
54.6	9.4	11.8	197
59.6	9.5	12.1	203
65.0	9.6	12.5	209
69.5	9.7	12.8	215
75.0	9.7	13.1	219
80.1	9.7	13.4	224

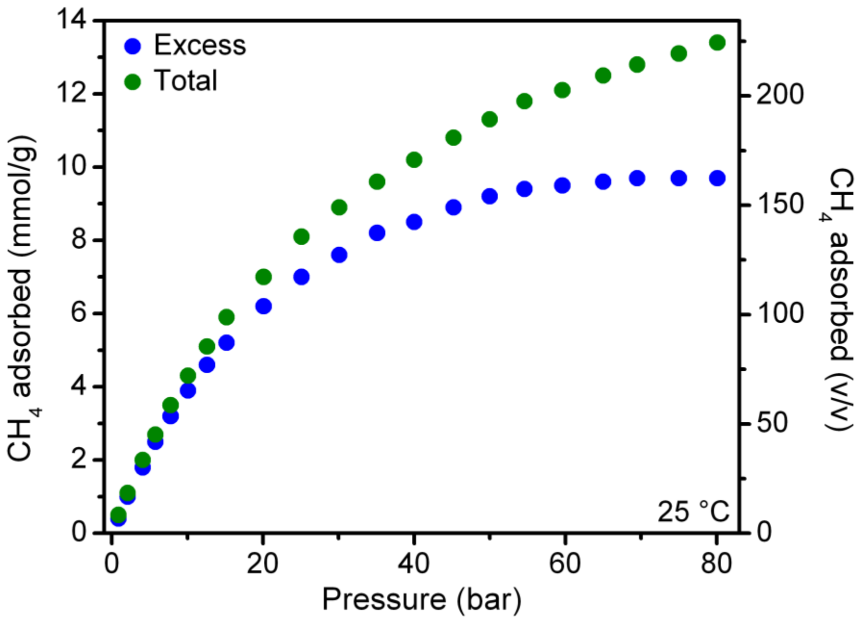

Figure S3. Excess and total CH4 adsorption isotherms for UiO-67-bpy at 25 °C.

Comparison to experiment: CH_4 adsorption of in MOF-5

In this section, we demonstrate the applicability of the methodology used in the main manuscript to predict reasonable usable capacities by comparing to experimental gas measurement results for MOF-5. The calculations for MOF-5 are performed on cluster models for the metal-cluster in MOF-5 and are shown in Figure S4. Model M2 is designed for studying adsorption on and near the face of the metal cluster ("cup-site"), by retaining the cluster itself and three coordinating linkers. The other three remote linkers are truncated by replacing their aromatic ring by methyl groups. Model M3 is designed for studying adsorption on the top of the metal cluster ("top-site"). It retains the metal cluster, and three relevant linkers while others are truncated by methyl groups.

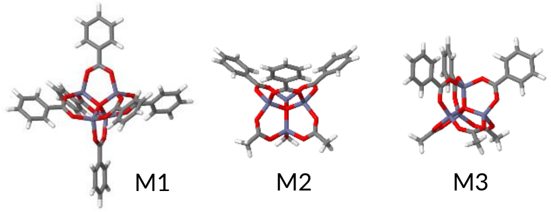

Figure S4. Models for MOF-5 adsorption sites. M1 represents the metal-cluster (Zn_4O^{6+}) coordinated by six linkers, represented by phenyl carboxylate ($C_7O_2H_6^-$). M2 and M3 are truncated versions of M1. M2 is used for studying CH_4 adsorption on the "cup-site" and M3 is used for the "top-site".

Model geometries are derived from the crystal structure obtained from the Cambridge Crystallographic Data Center, CSD entry SAHYIK01. Geometry optimization of the adsorbed molecules involves constraining the geometry of the cluster atoms to their crystalline locations, while optimizing the locations of the hydrogen atoms and the adsorbed CH_4 molecules. All calculations are performed using the B97M-V density functional.(7) Due to the large size of the cluster the basis set used for geometry optimization is 6-31g*, smaller than the basis used in the main-manuscript. Interaction energies are calculated using the def2-qzvp basis without counterpoise correction. The optimized geometries are verified to be minima on the potential energy surface using partial hessian analysis for the atoms of the adsorbed molecules. The calculated energies are shown in Table S4.

Table S4. Adsorption energies, in kJ/mol for the four different adsorption sub-sites on the cup-site.

	ΔE_{ads}	Multiplicity
c1	−18.9	1
c2	−12.1	3
c3	−12.0	3
t1	−12.0	1

Adsorption on the cup-site. It is found that there are three CH_4 adsorption sub-sites in the cup-site. The first sub-sites to be occupied, denoted by c1, is positioned above the center of the cluster. The second sub-site to be occupied, denoted by c2, is found between the two aromatic rings and is triply degenerate. The third sub-site, denoted by c3, is found above the aromatic rings and is also triply degenerate and are shared with adjacent metal-clusters. The cup-site can therefore adsorb up to seven CH_4 molecules when fully saturated.

Cup-site adsorption is a sequential, step-wise, reaction: for a CH_4 molecule to be adsorbed on c2, the c1 sub-site must first be occupied and for adsorption on c3, two adjacent c2 sub-sites must be occupied. This is a feature of the potential energy surface (PES) of the reaction: unless c1 is already adsorbed, c2 is not a minimum of the PES. Adsorption of a single CH_4 on c3 is not completely dependent on the presence of molecules in c2 and exists as a minimum of the PES in its absence with a relatively low ΔE_{ads}, if c1 is occupied.

Adsorption on the top-site. The "top-site" is located on the ZnO_4 cluster, above zinc atom and is can adsorb a single CH_4 molecule at t1.

Adsorption model. To connect the results above to experimentally measured adsorption isotherms, an adoption model is devised. The model assumes that a molecule cannot adsorbed on c2 unless c1 is also occupied. Also, a molecule cannot adsorb on a c3 sub-site, unless the two adjacent c2 sub-sites are also occupied.

The adsorption model is manifested by the adsorption polynomial, Q which represents the relative weight of each possible adsorption configuration. The form of Q is:

$$Q = K_{c1}K_{c2}^3K_{c3}^3p^7 + 3K_{c1}K_{c2}^3K_{c3}^2p^6 + 3K_{c1}K_{c2}^3K_{c3}p^5 + K_{c1}K_{c2}^3p^4 + 3K_{c1}K_{c2}^2K_{c3}p^4 + 3K_{c1}K_{c2}^2p^3 + 3K_{c1}K_{c2}p^2 + K_{c1}p + 1$$

The p^i term is the pressure of CH_4, raised to i'th power where i is the number of adsorbed molecules. The K_j terms are the equilibrium constants for configuration j and are evaluated using the well-known relation $K_j = \exp(-\Delta G_j/RT)$ where ΔG_j is the free energy of adsorption. If configuration K_j is degenerate, K_j is multiplied by the number of possible combinations c_j. The average number of CH_4 molecules that occupy the site at a given pressure, $\theta(p)$, is given by:

$$\theta(p) = Q^{-1}\sum_j iK_j\, p^i$$

The value of $\theta(p)$ can be as high as the maximal number of adsorbed molecules, which is six and half in this case in this case.

The CH_4 uptake at a given pressure, $n_{ads}(p)$, is given by:

$$n_{ads}(p) = \theta_{c1}(p)\cdot n_{1CH4} + \theta_{c2}(p)\cdot n_{1CH4} + \theta_{c3}(p)\cdot n_{1CH4}/2$$

where n_{sat} is maximal CH_4 uptake of a single adsorption sub-site. The contribution of the c3 sites, $\theta_{c3}(p)$, is halved since they are shared with adjacent metal clusters.

The value of n_{sat} is derived from the crystal structure, given that the concentration of a single adsorbed CH_4 is similar to the concentration of its adsorption site. The value of n_{sat} is found to be 70.38 v[STP]/v. The free energy of adsorption, ΔG_{ads}, is evaluated assuming a constant value of $\Delta S_{ads} = -9.5R$ which represents an characteristic value measured for materials used for adsorptive storage applications.(3,4) The enthalpy of adsorption, is evaluated as: $\Delta H_{ads} = \Delta E_{ads} - RT + E_{vib}$ where E_{vib} is the internal energy of a single vibration that is formed when a CH_4 molecule loses one of its translational degrees of freedom by being adsorbed to the surface.

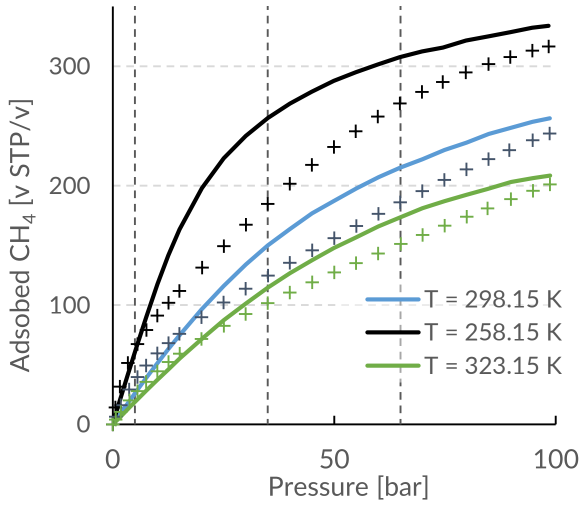

Figure S5. MOF-5 adsorption isotherms at various temperatures. Experimental and theoretical-model curves are marked by solid lines and crosses respectively.

A comparison of the experimental vs. model isotherms is shown in Figure S5. Considering that no parameters are fitted, the results are in good agreement with experiment. The model over-adsorbs in both higher and lower pressures, presumably due to inaccurate values of ΔS_{ads} which are too low. The actual values of ΔS_{ads} in c1 and c3 are expected to be higher than the value used, since the molecular motions are more restricted for molecules adsorbed on these sub-sites; c1 is tightly bonding with a relatively high ΔE_{ads} and motions in c3 are restricted due to the presence of two near neighbors in adjacent c2 sub-sites.

At lower temperature (T=253.15 K) the predicted isotherm underestimates CH_4 uptake at the range of approximately 5 to 50 bar, presumably due to increasing importance of lower adsorption-energy sub-sites on the linkers, which are not accounted for in this model. Also, stand-alone adsorption on c3, with no adjacent molecules in c2 might become significant. Lastly, ΔS_{ads} is possibly temperature dependent and can increase at lower temperatures.

Usable capacity. A comparison of the usable capacities obtained by the model vs. experimental results for MOF-5 are shown in Table S5. The usable capacity is defined as:

$$n_{usable} = \theta(p_{max} - p_{min}) \times n_{sat}$$

This is equivalent to the difference in the amount of CH_4 adsorbed at high pressure (35 or 65 bar) minus the minimal pressure of 5.8 bar. At P_{max} = 35 bar the model provides a qualitative level of agreement, while better accuracy is achieved for P_{max} = 65. Presumably, the main origin of error is the model's tendency to over-bind at lower pressures.

Table S5. Usable capacity of MOF-5. Comparison between theory and experiment.

		Temp. [K]	Exp. [v/v]	Model [v/v]	% Err.
Pmin=5.8 bar	Pmax=35 bar	298.15	121	85	-30%
		258.15	190	117	-48%
		311.15	106	79	-26%
		323.15	94	74	-21%

		Temp. [K]	Exp. [v/v]	Model [v/v]	% Err.
Pmin=5.8 bar	Pmax=65 bar	298.15	186	146	-22%
		258.15	279	229	-18%
		311.15	165	126	-23%
		323.15	145	111	-23%

References

1. Li H, Eddaoudi M, O'Keeffe M, Yaghi OM. Design and synthesis of an exceptionally stable and highly porous metal-organic framework. Nature. 1999 Nov 18;402(6759):276–9.

2. Li L, Tang S, Wang C, Lv X, Jiang M, Wu H, et al. High gas storage capacities and stepwise adsorption in a UiO type metal–organic framework incorporating Lewis basic bipyridyl sites. Chem Commun. 2014 Feb 4;50(18):2304–7.

3. Mason JA, Veenstra M, Long JR. Evaluating metal–organic frameworks for natural gas storage. Chem Sci. 2013 Nov 26;5(1):32–51.

4. Bhatia SK, Myers AL. Optimum Conditions for Adsorptive Storage. Langmuir. 2006 Jan;22(4):1688–700.

5. Gonzalez MI, Bloch ED, Mason JA, Teat SJ, Long JR. Single-Crystal-to-Single-Crystal Metalation of a Metal–Organic Framework: A Route toward Structurally Well-Defined Catalysts. Inorg Chem. 2015 Mar 16;54(6):2995–3005.

6. Lemmon E, McLinden M, Huber M. REFPROP: Reference fluid thermodynamic and transport properties. NIST Stand Ref Database. 2007;23(8.0).

7. Mardirossian N, Head-Gordon M. Mapping the genome of meta-generalized gradient approximation density functionals: The search for B97M-V. J Chem Phys. 2015 Feb 21;142(7):074111.
